# FGL1-mediated lymph node metastasis in stage T1 non-small cell lung cancer: therapeutic targeting

**DOI:** 10.1186/s40164-025-00709-5

**Published:** 2025-09-29

**Authors:** Xi-Yang Tang, Run-Ze Zhang, Zhi-Bo Feng, Yu-Long Zhou, Wei-Guang Du, Chen Shu, Yang Shen, Meng-Chao Li, Jun-Chao Cai, Xiao-Long Yan, Nan Ma, Jin-Bo Zhao

**Affiliations:** 1 Department of Thoracic Surgery, Tangdu Hospital, Fourth Military Medical University, 569 Xinsi Road, Xi’an, 710038 Shaanxi China; 2 Department of Ophthalmology, Tangdu Hospital, Fourth Military Medical University, 569 Xinsi Road, Xi’an, 710038 Shaanxi China; 3 Department of Immunology, Zhongshan School of Medicine, Sun Yat-Sen University, Guangzhou, 510515 China

**Keywords:** N2 lymph node metastasis, T1 NSCLC, FGL1, CCNE1(+) cells, Single-cell sequencing, ShFGL1_ AAV6, ShFGL1_ AAV9

## Abstract

**Background:**

Approximately 30% of patients with stage T1 non-small cell lung cancer (NSCLC) have mediastinal (N2) lymph node metastasis; however, the underlying mechanism remains unclear.

**Methods:**

The cells likely mediating N2 lymph node metastasis in T1 NSCLC were identified by single-cell sequencing. The expression and function of the main functional gene high fibrinogen-like protein 1 (FGL1) in this cell subgroup were analyzed by single-cell analysis. Transcriptome sequencing, metabolome sequencing, and mass spectrometry combined with in vitro and in vivo experiments were conducted, and therapeutic validation was performed using shFGL1_AAV9 and shFGL1_AAV6.

**Results:**

A novel cell subgroup characterized by FGL1 expression was identified (CCNE1(+) cells). FGL1 expression coincided with the appearance of this cell subgroup, suggesting that FGL1 + cells mediate T1 NSCLC N2 lymph node metastasis. Mass spectrometry combined with transcription sequencing and metabonomics revealed that FGL1 may affect glycolysis regulators and participate in epithelial-to-mesenchymal transition in NSCLC via the PI3K/AKT/HIF-1α pathway. Further analyses suggested that FGL1 promotes tumor proliferation, metastasis, and lymph tube formation, ultimately inducing lymph node metastasis. This was verified in vivo and in vitro. FGL1 knockdown inhibited these processes. Finally, shFGL1_AAV9 and shFGL1_AAV6 were verified as novel targeted therapies to knock down FGL1 in vivo, supporting the identification of new therapeutic targets to inhibit NSCLC metastasis.

**Conclusion:**

We elucidated the role of FGL1 in NSCLC, proposing that FGL1 acts like a “shield machine cutter” in mediating T1 NSCLC N2 lymph node tube formation, creating metastasis channels. This provides the basis for novel FGL1-targeting treatment strategies.

**Graphical abstract:**

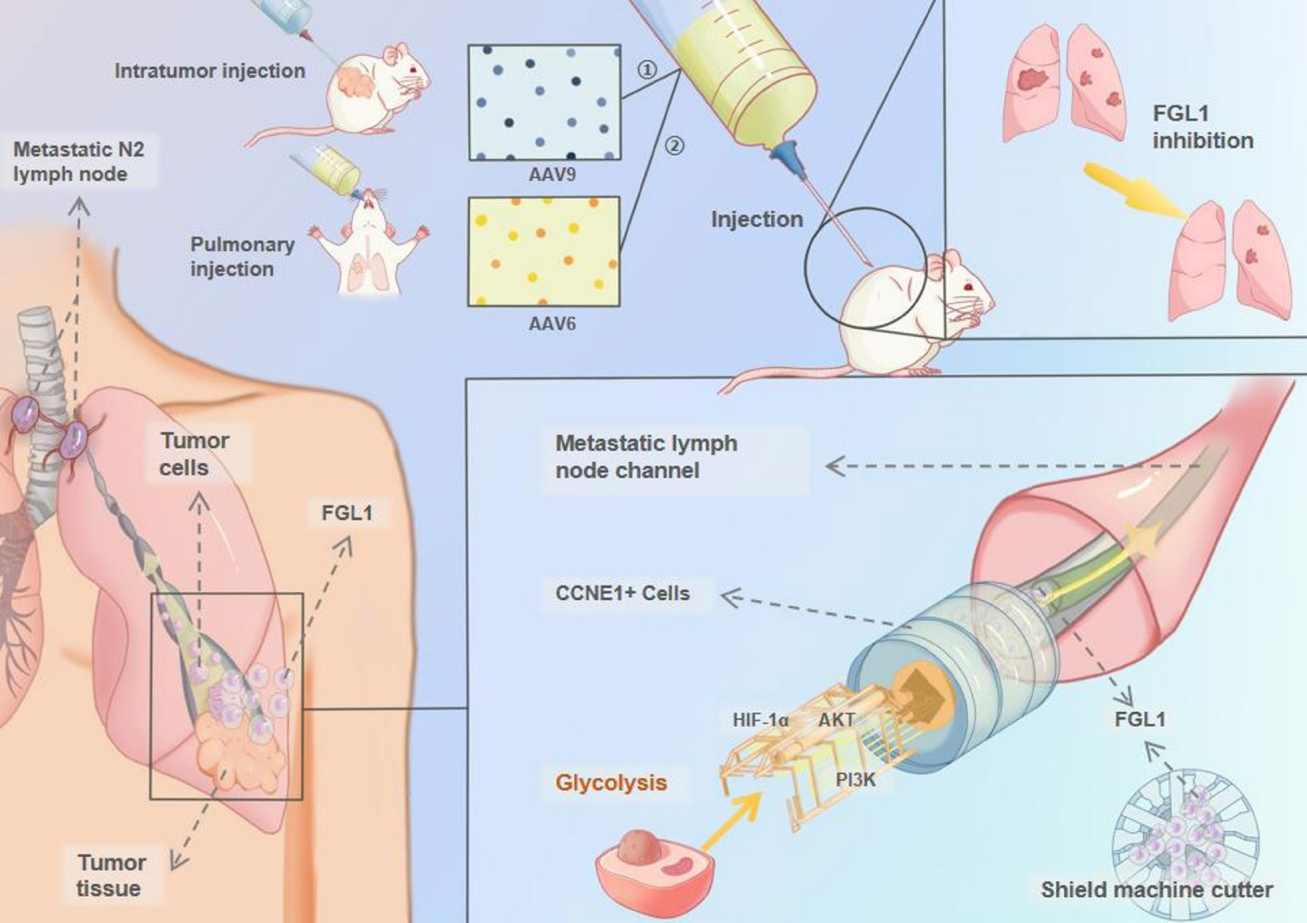

**Supplementary Information:**

The online version contains supplementary material available at 10.1186/s40164-025-00709-5.

## Introduction

Lung cancer is the tumor with the highest morbidity and mortality [1, 2]. Early non-small cell lung cancer (NSCLC), especially stage I NSCLC with a tumor body size ≤ 3 cm (T1) and no metastasis (N0M0), has a high cure rate. However, clinical research shows that approximately 30% of patients with stage T1 NSCLC will have mediastinal (N2) lymph node and distant organ (M1) metastasis [3].

Recent studies on the molecular mechanisms of tumor lymph node metastasis focuses on three main aspects: the metabolic adaptation of tumor cells to the lymph node microenvironment [4], the interaction between chemokine receptors on cancer cells and ligands in lymph nodes, key immunogenomic patterns associated with lymph node metastasis [5, 6], and the direct promotion of lymphangiogenesis by tumors [7]. However, current research investigating the mechanism of lymph node metastasis or recurrence in T1 stage tumors is limited. Most existing studies focus on the risk factors and prediction of lymph node metastasis and recurrence in T1 stage Tumors. For example, pro-surfactant protein B has been strongly associated with the recurrence of early-stage NSCLC, and its downregulation induces the recurrence of NSCLC by activating phosphoglycerate kinase 1-mediated AKT signaling [8]. Deep submucosal invasion has been identified as an independent risk factor for lymph node metastasis of stage T1 colorectal cancer and can be predicted using nomogram models [9, 10]. Similar methods have also been employed in studies of stage T1 breast cancer [11].

Nevertheless, the molecular mechanism of lymph node metastasis in T1 stage tumors and the mechanism underlying N2 lymph node metastasis in stage T1 NSCLC remain unclear. Immune checkpoints contribute to the malignant progression of tumor by regulating tumor immune microenvironment and promote malignant behaviors such as tumor cell proliferation and metastasis [12], including programmed cell death 1 (PD-1) [13–15], poliovirus receptor (PVR) [16, 17], SIRPA [18, 19] and other immune checkpoints. However, their role in mediating N2 lymph node metastasis in stage T1 NSCLC remains unclear. Therefore, elucidating the underlying mechanisms of N2 lymph node metastasis in stage T1 NSCLC is of significant clinical importance for the diagnosis, treatment, and prognosis monitoring of affected patients.

**Fig. 1:**
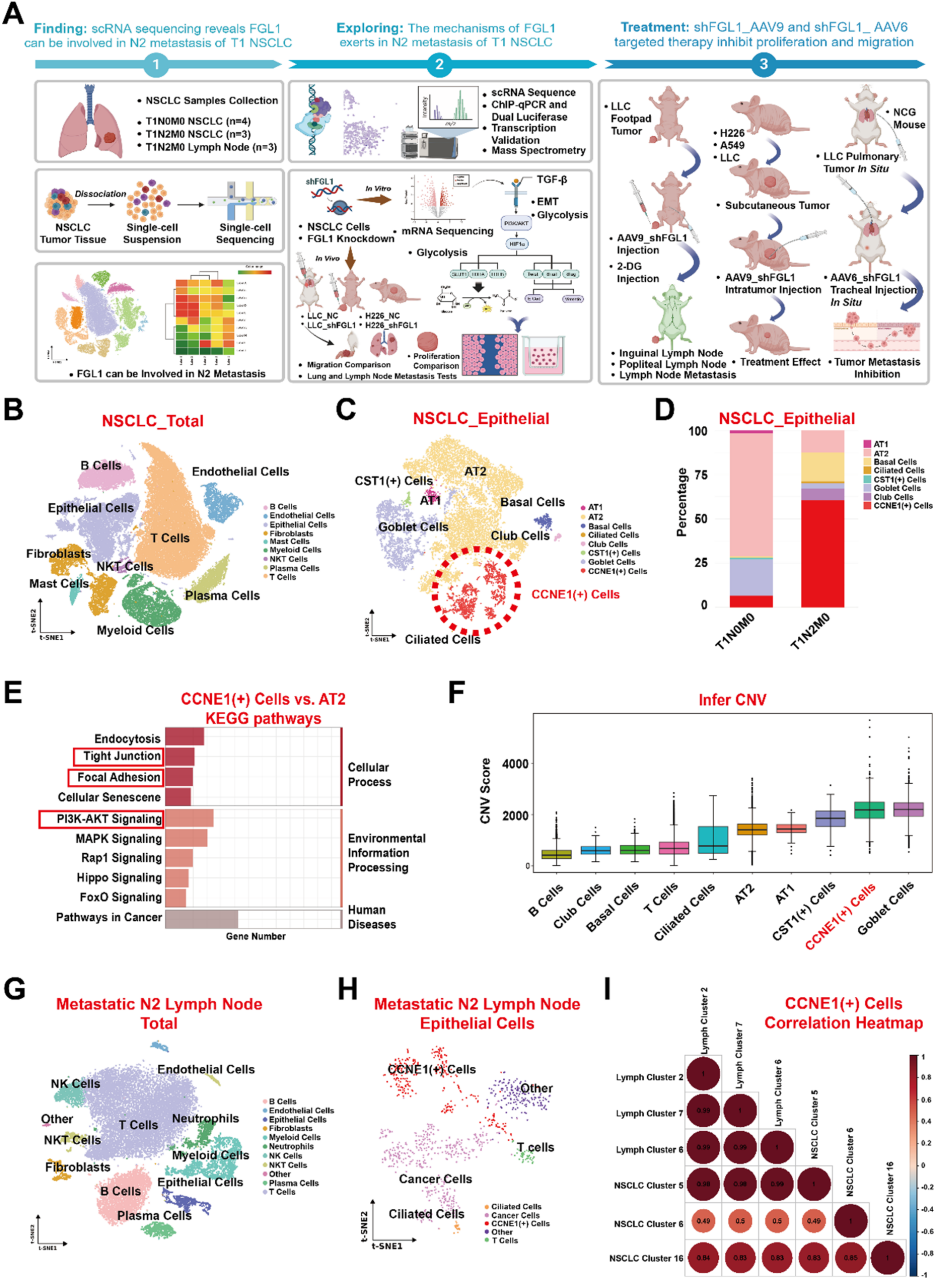
Single-cell sequencing reveals a novel cell subgroup mediating lymph node metastasis. **A** Strategy map of the study. **B** Overall cluster map from scRNA-seq of seven NSCLC samples. **C** Subgroup of epithelial cells in these seven NSCLC samples. **D** Cell proportion analysis of epithelial cells. **E** KEGG analysis was used to compare CCNE1(+) Cells with AT2 cells, and differential gene analysis was performed. **F** CNV analysis of epithelial cell subgroups was performed to evaluate epithelial subgroup cell malignancies. **G** Overall cluster map from scRNA-seq of three metastatic N2 lymph node samples. **H** Subgroup of epithelial cells in these three metastatic N2 lymph node samples. **I** Correlation between clusters of all CCNE1(+) Cells in NSCLC and metastatic N2 lymph node samples

In this study, single-cell RNA sequencing (scRNA-seq) was used to analyze T1N0M0 and T1N2M0 NSCLC tissue samples, as well as N2 lymph node metastasis samples, identifying a novel cell cluster known as CCNE1(+) cells. We found high fibrinogen-like protein 1 (FGL1) expression in CCNE1(+) cells and hypothesized that FGL1 might be involved in N2 lymph node metastasis in stage T1 NSCLC, acting like a “cutter head” of a shield machine. To verify this theory, we conducted a series of in vitro and in vivo experiments to prove that FGL1 facilitates glycolysis and epithelial-mesenchymal transition (EMT) through the phosphatidylinositol 3-kinase (PI3K)/AKT/hypoxia-inducible factor-1α (HIF-1α) pathway, thereby enhancing tumor metastasis. We found that shFGL1_AAV6 and shFGL1_AAV9 targeted therapies showed promising therapeutic effects in inhibiting tumor proliferation and metastasis. Figure 1A shows the study design. This study proposes a mechanism for FGL1-mediated N2 lymph node metastasis in stage T1 NSCLC and a therapy strategy targeting FGL1.

## Materials and methods

### Mice

Four-week-old male BALB/c nude (BALB/cJGpt-Foxn1nu/Gpt) and NCG (NOD/ShiLtJGpt-Prkdc^em26^Il2rg ^em26^/Gpt) mice were purchased from GemPharmatech Co.,Ltd (Nanjing, China). All in vivo experiments were reviewed and approved by the Ethics Committee of the Air Force Medical University (No. 20220666).

### Cell lines

All cell lines were purchased from Wuhan Pricella Biotechnology Co., Ltd. (Wuhan, China) H226, H1299, and H1703 cells were cultured in Gibco RPMI-1640 medium (Cat# 11875119, Thermo Fisher Scientific, Waltham, MA, USA) Supplemented with 10% fetal bovine serum. A549 cells were cultured in Ham’s F-12 K medium (Cat# PM150910B, Pricella, China) Supplemented with 10% fetal bovine serum (Cat# BS-1101, OPCEL, China). Lewis lung carcinoma (LLC) cells were cultured in Dulbecco’s modified Eagle medium (high-sugar; Cat# PM150210, Pricella, China) Supplemented with 10% fetal bovine serum. All cell cultures were maintained at 37 °C in a 5% CO2 atmosphere.

### NSCLC samples collection

This study collected a total of 316 NSCLC tissue samples (including 158 lung adenocarcinoma, 133 lung squamous cell carcinoma, and 25 adenosquamous carcinoma cases) with paired paracancerous tissues, along with an additional 15 T1N0M0 and 15 T1N2M0 NSCLC tissue samples from surgical patients at Tangdu Hospital (Xi’an, China). The eligible patients provided written informed consent, giving consent for the collection and use of their tissue samples and other clinical data. All experiments performed using these samples were approved by the Ethics Committee of the Air Force Medical University (No. 202203-040).

### Immunohistochemistry and immunofluorescence

The immunohistochemistry (IHC) and immunofluorescence (IF) staining protocols used were performed as previously described [20]. Anti-FGL1 polyclonal antibody (Cat# 16000-1-AP, Proteintech, China), anti-Cyclin E1 polyclonal antibody (Cat# 40804, Signalway Antibody, China), glucose transporter (GLUT)1 polyclonal antibody (Cat# 40967, Signalway Antibody, China), GLUT3 Rabbit polyclonal antibody (Cat# 55439, Signalway Antibody, China), phosphoglycerate mutase 1 (PGAM1) Rabbit monoclonal antibody (mAb) (Cat# 49976, Signalway Antibody, China), lactate dehydrogenase A (LDHA) antibody (Cat# 48492, Signalway Antibody, China), lactate dehydrogenase B (LDHB) antibody (Cat# 48009, Signalway antibody, China), pyruvate dehydrogenase kinase-1 (PDK1) Rabbit mAb (Cat# 49573, Signalway antibody, China), Hexokinase 2 (HK2) antibody (Cat# 32115, Signalway Antibody, China), transforming growth factor-beta (TGF-β) (Cat# 21898-1-AP, Proteintech, China), Twist (Cat# 25465-1-AP, Proteintech, China), Slug (Cat# 53574, Signalway Antibody, China), Snail (Cat# 54899, Signalway Antibody, China), Vimentin (Cat# 10366-1-AP, Proteintech, China), and E-cadherin (Cat# 60335-1-Ig, Proteintech, China) were used in the study.

### Chromatin immunoprecipitation-quantitative polymerase chain reaction (ChIP-qPCR)

A chromatin immunoprecipitation **(**ChIP) kit (Cat# Bes5001, BersinBio, China) was used with a specific anti-FGL1 antibody (Cat# 16000-1-AP, Proteintech, China). Quantitative polymerase chain reaction (qPCR) was used to amplify immunoprecipitated DNA. The E26 transformation-specific transcription factor 1 (*ETS1*) and *FGL1* promoter region primer 1 sequences were as follows:

AATAATGTTACTCCAAGAAAATTTGGCTTTTGGAATTCCTCCACAAAGTATTGGCCCTAGAGTAGTCCTTACTGCCTGATTAACTTTTGCAAGTTTCTCCTGAGGTAATGATGCTAGCCAAGTTTCCGAAAGACAGCATAGTCTACCTTTAGTAGAAAAGAGGACAGATTCTGAGCAGGATTGCCTTTGAGCCCAGCTATACCTCTTGTTAGCTGTGTGACCTCGTGCAAGTCACTTAAACCTCTGTGTCTCAGTTTCGTCATTTGTGAAATGGAGATCATTAACAGTACCTACTTATAG.

The *FGL1* promoter region primer 3 sequence was:

TATAGGGCTGTGTGGCTGAGTGAGTTGATAAAATTACCATGTTTAGCACATGATAAGTATACCACATGTTAGCTGCTGTTAATGTTATGTCCCTCCAGTGAGGAAATGAACACAAGGCTAAATTATTGCTTGAATGGGGACAAAGCTAAAGGCATACAAGAAGGAAAGATAAGTTTGTATTTCAAAATATGTCATTTTACCACAGAGATCATTATTGAATTAGCCGAATAATCCCATATTTTTGCTTTTGAAATTTAACATTAGCAGCTTGAATTCTCTACAATTATGAATGTATAATTT.

### Luciferase reporter assay

Three ETS1–*FGL1* binding sites were mutated in the *FGL1* promoter. Invitrogen Lipsome 2000 (Cat# 11668019, Thermo Fisher Scientific, China) was used for transfecting FGL1-promoter-WT/Renilla and FGL1-promoter-MT/Renilla plasmids into 293 T cells. A Dual-Glo Luciferase Assay Kit (Cat# E1910, Promega, China) was used to measure the *FGL1* promoter activity.

### Immunoprecipitation and mass spectrometry

FGL1 was collected using a total protein collection kit (Cat# SD-001/SN-002, Invent Biotechnologies, China) following the manufacturer’s instructions. Briefly, the SD-001 buffer was used for cell lysis on ice for 10 min, and the solution was centrifuged for 30 s at 12,000 rpm. The sample was incubated with the FGL1 immunoprecipitation antibody for 8 h at 4 ℃, and protein A/G agarose was Subsequently incubated for 2 h. Samples were collected and analyzed using mass spectrometry by Beijing Tsingke Biotechnology Co., Ltd (Beijing, China).

### FGL1 knockdown

*FGL1* was knocked down in A549, H226, H1299, H1703, and LLC cell lines using targeted short hairpin RNAs (shRNAs) synthesized by HanBio Therapeutics (Shanghai, China). Polybrene (2 mg/mL) was used for shRNA transfection. When the cell density reached 70% in a 6-well plate, polybrene was used for transfection for 24 h, followed by purinomycin for cell screening. The sequence for the *FGL1* knockdown has been reported in our previous study [21].

### Single-cell sequencing and analysis

Ten NSCLC samples, including T1N0M0 (*n* = 4), T1N2M0 (*n* = 3), and three metastatic N2 lymph node samples from patients with T1N2M0, were obtained for scRNA-seq. All samples were dissociated into single-cell Suspensions. Large population and Subpopulation cell identification, as well as 10× Genomics scRNA-seq and analysis, were performed by LC BioTechnology Co., Ltd (Hangzhou, China).

### RNA-sequencing and differential expression analysis

H226/A549 cells (1 × 10^7^ cells) were seeded, and total RNA was extracted using TRIzol (Cat# MF035-01, Mei5bio, China). RNA samples were collected for RNA-seq analysis. The collected data were further assessed using Kyoto Encyclopedia of Genes and Genomes (KEGG), Gene Ontology (GO), and Gene Set Enrichment Analysis (GSEA) to determine differential gene expression. RNA-seq and analysis of H226 cells were conducted by LC BioTechnology Co., Ltd (Hangzhou, China). A549 cell sequencing and analysis were conducted by Beijing Tsingke Biotechnology Co., Ltd (Beijing, China).

### Metabonomic analysis

A549 cells (1 × 10^7^ cells) were collected and the pellet was processed for untargeted metabolomic sequencing by Beijing Tsingke Biotechnology Co.,Ltd. In depth analysis of differential metabolites was conducted using KEGG, GO, and GSEA.

### Western blotting

Western blotting was performed to explore protein expression changes following *FGL1* knockdown, focusing on markers related to EMT, glycolysis, and the PI3K/AKT signal-related pathways. Primary antibodies used in this study are as follows: TGF-β (Cat# 21898-1-AP, Proteintech, China), Twist (Cat# 25465-1-AP, Proteintech, China), Slug (Cat# 53574, Signalway Antibody, China), Snail (Cat# 54899, Signalway Antibody, China), Vimentin (Cat# 10366-1-AP, Proteintech, China), E-cadherin (Cat# 60335-1-Ig, Proteintech, China), HIF-1α (Cat# 41005, Signalway Antibody, China), PDK1 (Cat# 49573, Signalway Antibody, China), GLUT1 (Cat# 40967, Signalway Antibody, China), GLUT3 (Cat# 55439, Signalway Antibody, China), HK2 (Cat# 32115, Signalway Antibody, China), PGAM1 (Cat# 49976, Signalway Antibody, China), LDHA (Cat# 48492, Signalway Antibody, China), LDHB (Cat# 48009, Signalway Antibody, China), dihydrolipoamide dehydrogenase (DLDH) (Cat# 56779, Signalway Antibody, China), AKT (Phospho-Ser473) (Cat# 11054, Signalway Antibody, China), AKT (Phospho-Thr308) (Cat# 11055, Signalway Antibody, China), AKT (Cat# 33748, Signalway Antibody, China), PI3K (Cat# 41337, Signalway Antibody, China). Western blotting was performed as previously reported [22].

### Glucose consumption, lactate production, and pyruvate production assays

Cells were seeded in a 24-well plate (3 × 10^5^ cells/well) with 300 µL of culture medium per well and cultured for 24 h. The culture Supernatant was collected and centrifuged at 3000 rpm for 10 min. Glucose, lactate, and pyruvate were detected using their respective assay kits (Nanjing Jiancheng Bioengineering Institute, China) following the manufacturer’s protocols.

### Lymph tube formation assay

Polyl-lysine solution (Cat# PB180522, Pricella, China)-coated 24-well plates were prepared, and 150 µL of matrigel matrix (Cat# 354234, Corning, China) was added to each hole and incubated at 37 °C for 30 min. In total, 50,000 lymphatic endothelial cells (Cat# CP-H026, Pricella, China) in the lower chamber were placed in 600 µL medium, and 50,000 A549/H1703 negative control (NC) or A549/H1703 shFGL1 cells in the upper chamber were added to 200 µL medium for indirect co-culture. Lymphatic vessel formation was observed.

### Tumor proliferation assays

A549, H226, H1299, and H1703 cell Lines were centrifuged at 1,000 rpm for 5 min, and 1,000 cells were seeded in sextuplicate per cell Line in 96-well plates in 100 µL of RPMI-1640 medium. After 48 h of culture, the medium was replaced with medium containing 9 mM of 2-Deoxy‐D‐glucose (2-DG) in the 2-DG (Cat# T6742, TargetMol, China) treatment group, while the control group received normal culture medium. Cell counting kit-8 (CCK-8) (Cat# C6005, New Cell & Molecular Biotech Co. Ltd, China) was used according to the manufacturer’s instructions, and cell density was measured at 450 nm.

An additional 1,000 cells were seeded in 6-well plates and cultured for 2 weeks for colony formation tests. The medium in the 2-DG treatment group was replaced with medium containing 9 mM of 2-DG following a 4-day incubation period, while the control group received a fresh Supply of standard culture medium. Upon completion of the culture, the 6-well plates were washed two times with phosphate-buffered saline, fixed with paraformaldehyde for 20 min, and stained with 1% crystal violet for 30 min.

### Tumor migration assays

A549, H226, H1299, and H1703 cells were seeded on 10 cm plates, scratched when cell density reached 80–90%, and observed for 48 h. After scratch formation, the 2-DG treatment group was switched to a medium containing 9 mM of 2-DG, while the control group was switched to normal culture medium.

For the transwell assay, 70,000 cells were collected and added to the upper chamber of a transwell plate, 200 µL of serum-free RPMI-1640 medium (Supplemented with 10 µL of 180 mM 2-DG for the 2-DG treatment group) was added, and 600 µL of 20% serum RPMI-1640 medium (Supplemented with 30 µL of 180 mM 2-DG for the 2-DG treatment group) was added to the lower chamber. After 24–48 h, the cells were fixed with paraformaldehyde for 20 min and stained with 1% crystal violet for 30 min.

### Xenograft tumor experiments

Male mice (BALB/cJGpt-Foxn1nu/Gpt; 4 weeks old; 15–20 g) were housed in a specific pathogen-free house for 1 week. They were randomly assigned to two groups and injected Subcutaneously in the anterior Limb with 8× 10^6^ H226 or A549 cells, or 8 × 10^5^ LLC cells that had been modified to express FGL1 (FGL1_NC) or not (FGL1_knockdown (KD)). The tumor volume (mm^3^) was measured every 2–3 days as (tumor length [mm] × square of tumor width [mm]^2^)/2.

A total of 8 × 10^5^ LLC cells that had been modified to express FGL1 or not were injected into the foot pad of male mice (BALB/cJGpt-Foxn1nu/Gpt; 4 weeks old; 15–20 g). Tumor formation and metastasis were assessed using small animal imaging after 14 days. Subsequently, the inguinal and popliteal lymph nodes were taken, and the lymph node metastasis was observed by hematoxylin and eosin (HE) staining.

### LLC cell in situ lung injection and shFGL1-AAV6 non-invasive tracheal injection

Four-week-old male NCG (NOD/ShiLtJGpt-Prkdc^em26^Il2rg^em26^/Gpt) mice were anesthetized, their skin was cut open, and 5 × 10^5^ LLC cells (with a luciferase tag) were Suspended in 100 µL of phosphate-buffered saline. The pleura and Subpleural lung tissue were exposed by carefully dissecting through the intercostal muscle. A 1-mL syringe was subsequently used to perform in situ injections directly into the lungs of the NCG mice.

Tumor formation in the mice was observed using small animal imaging, and shFGL1_AAV6 treatment was administered 2 days later. Mice were anesthetized with isoflurane and Suspended vertically using a thin thread tied to the upper teeth. A Suitably sized catheter was inserted into the trachea. Subsequently, 50 µL of HBAAV2/6-h-FGL1 shRNA1_EGFP (1.2 × 10^12^ vg/mL, synthesized by HanBio Therapeutics) was delivered into the catheter and slowly inhaled by the mouse into the lung.

### shFGL1_AAV9 intratumor injection targeted therapy

Male mice (BALB/cJGpt-Foxn1nu/Gpt; 4 weeks old; 15–20 g) were housed in a specific pathogen-free house for 1 week. They were randomly assigned to two groups and 8 × 10^6^ H226 or A549 cells, or 8 × 10^5^ LLC cells were injected subcutaneously into the anterior limb. HBAAV2/9-h-FGL1 shRNA1_EGFP (1.2 × 10^12^ vg/mL, synthesized by HanBio Therapeutics) was injected (40 µL) directly into the Tumor at multiple points when it reached 100–150 mm^3^. The rate of Tumor growth was evaluated and compared between the groups until it reached 1,000 mm^3^. When the Tumor volume of the control group reached 1,000 mm^3^, the tumor was surgically removed, and IHC was performed. The effectiveness of the targeted therapy in inhibiting tumor proliferation was subsequently evaluated.

Similarly, male mice (BALB/cJGpt-Foxn1nu/Gpt; 4 weeks old; 15–20 g) were ready, and 8 × 10^5^ LLC cells were injected into the foot pad. shFGL1_AAV9 was injected (20 µL) into the foot pad after Tumor formation once every 4 days, and 2-DG was administered intraperitoneally once every 2 days at 1800 mg/kg. The combination therapeutic effect was observed using small animal imaging, and the inguinal and popliteal lymph nodes were taken after 14 days. Finally, lymph node metastasis was observed using HE staining.

### Quantification and statistical analysis

Data are presented as mean ± standard error of the mean. Statistical analyses and graph generation were performed using GraphPad Prism 8.0 (GraphPad Software, USA). ImageJ (National Institutes of Health) was used for quantification and cell counting. Unpaired *t*-tests were used to assess biological triplicate samples in the in vitro and in vivo studies. The Wilcoxon signed-rank test was used when the sample size was > 60.

## Results

### Single-cell sequencing reveals a novel cell subgroup CCNE1(+) cells mediating lymph node metastasis

A novel group of cells known as CCNE1(+) Cells was identified as a potential driver of lymphatic metastasis in stage T1 NSCLC. Seven NSCLC samples were collected for 10× Genomics scRNA-seq, including T1N0M0 (*n* = 4) and T1N2M0 (*n* = 3). A total of 72,401 cells were categorized into the following nine subgroups: B, endothelial, epithelial, fibroblast, mast, myeloid, natural killer (NK) T (NKT), plasma, and T cells (Fig. 1B). Among them, 5,983 B cells were further classified into memory cell, naïve cell, plasma cell, and plasmacytoid dendritic cell (DC) (Supplementary Fig. 1A); 7,071 myeloid cells were categorized into cDC_type 1, cDC_type 2, cDC_type 3, M1, and M2 macrophages, and neutrophils (Supplementary Fig. 1B); 6,271 fibroblasts were divided into antigen-presenting cancer-associated fibroblasts (CAFs), CAFs, inflammation-associated fibroblasts, myofibroblasts, normal fibroblasts, proliferative fibroblasts, and pericytes (Supplementary Fig. 1C); 3,278 endothelial cells (ECs) were classified in alveolar, extra-alveolar, arterial, lymphatic, tumor, and venous ECs (Supplementary Fig. 1D); and 29,131 T cells were categorized into effector CD8^+^, follicular CD4^+^, naïve CD4^+^, regulatory (Treg) CD4^+^, tissue-resident memory CD4^+^, tumor Treg, and γδ T cells (Supplementary Fig. 1E).

CCNE1(+) Cells were most prevalent in T1N2M0 NSCLC samples with the highest degree of malignancy. Epithelial cells (14,232) were classified into the following eight subgroups: alveolar type 1 (AT1), AT2, basal, ciliated, club, cystatin-SN(CST1+), goblet, and CCNE1(+) Cells (Fig. 1C). Among them, the contents of clusters 5, 6, and 16 were significantly increased in T1N2M0 samples. Based on their high and specific expression of the CCNE1 gene, which plays an important role in promoting the malignant progression of tumors [23], we defined this group of cells as CCNE1(+) Cells. Generally, the marker genes of CCNE1(+) Cells were identified as *CENPF*, *CCNE1*, *STMN1*, *TOP2A*, *TPX2*, and *PBK* (Supplementary Fig. 1F) and exclusively existed in Epithelial cells (Supplementary Fig. 1G). Cell proportion analysis showed a higher proportion of CCNE1(+) Cells in T1N2M0 samples than in T1N0M0 samples (Fig. 1D). To better understand the function of this group of cells, KEGG analysis was used to compare CCNE1(+) Cells with AT2 cells and differential gene analysis was performed. The cells were mainly enriched in cell adhesion-related pathways such as tight junctions and focal adhesions, as well as in classical tumor progression pathways including the PI3K/AKT signaling pathway (Fig. 1E). Malignancy degree analysis using Infer copy number variation (CNV) was performed to evaluate epithelial subgroup cell malignancies, and CCNE1(+) Cells were found with a high CNV score (Fig. 1F).

The CCNE1(+) Cells in the lung cancer and metastatic lymph nodes were identical. Three metastatic lymph nodes from patients with T1N2M0 were collected for scRNA-seq. A total of 23,950 cells were classified into the following 10 subgroups: B, endothelial, epithelial, fibroblast, neutrophils, myeloid, NK, NKT, plasma, and T cells (Fig. 1G). Among them, 982 epithelial cells were primarily categorized into three subgroups as follows: CCNE1(+), cancer, and ciliated cells (Fig. 1H). Copycat and Infer CNV were performed to evaluate the malignancy of the epithelial subgroup cells, and the results were the same as those for NSCLC. CCNE1(+) Cells were the malignant subgroup of epithelial cells in metastatic N2 lymph nodes (Supplementary Fig. 2F, G). Correlation coefficient analysis was conducted according to the similarity of cell gene expression levels to verify whether CCNE1(+) Cells in metastatic N2 lymph nodes and NSCLC samples are identical. The correlation between clusters of most CCNE1(+) Cells was > 0.9, indicating that the same group of cells was present in NSCLC and metastatic N2 lymph nodes. This demonstrated that these cells were key mediators of N2 lymph node metastasis in stage T1 NSCLC (Fig. 1I). Clustering of other cells, including B cells, myeloid cells, fibroblasts, ECs, and T cells, was observed (Supplementary Fig. 2A−E).

We also validated the existence of CCEN1(+) Cells in nine lung cancer-related samples, including normal tissues (two-stage IA and one-stage IIIA lung adenocarcinoma) and three-stage IA and IIIA lung adenocarcinoma tissues, from the public database GSE 131,907 and dataset 10.24433CO.0121060.v1 [24]. The results in the public dataset confirmed the higher proportion of CCNE1(+) Cells in stage IIIA lung cancer, which were associated with metastasis and glycolysis (Supplementary Fig. 3A–F).

### FGL1 is highly expressed in CCNE1(+) cells, its appearance coincides with their appearance, and it has an independent role in promoting metastasis in addition to immune regulation

**Fig. 2:**
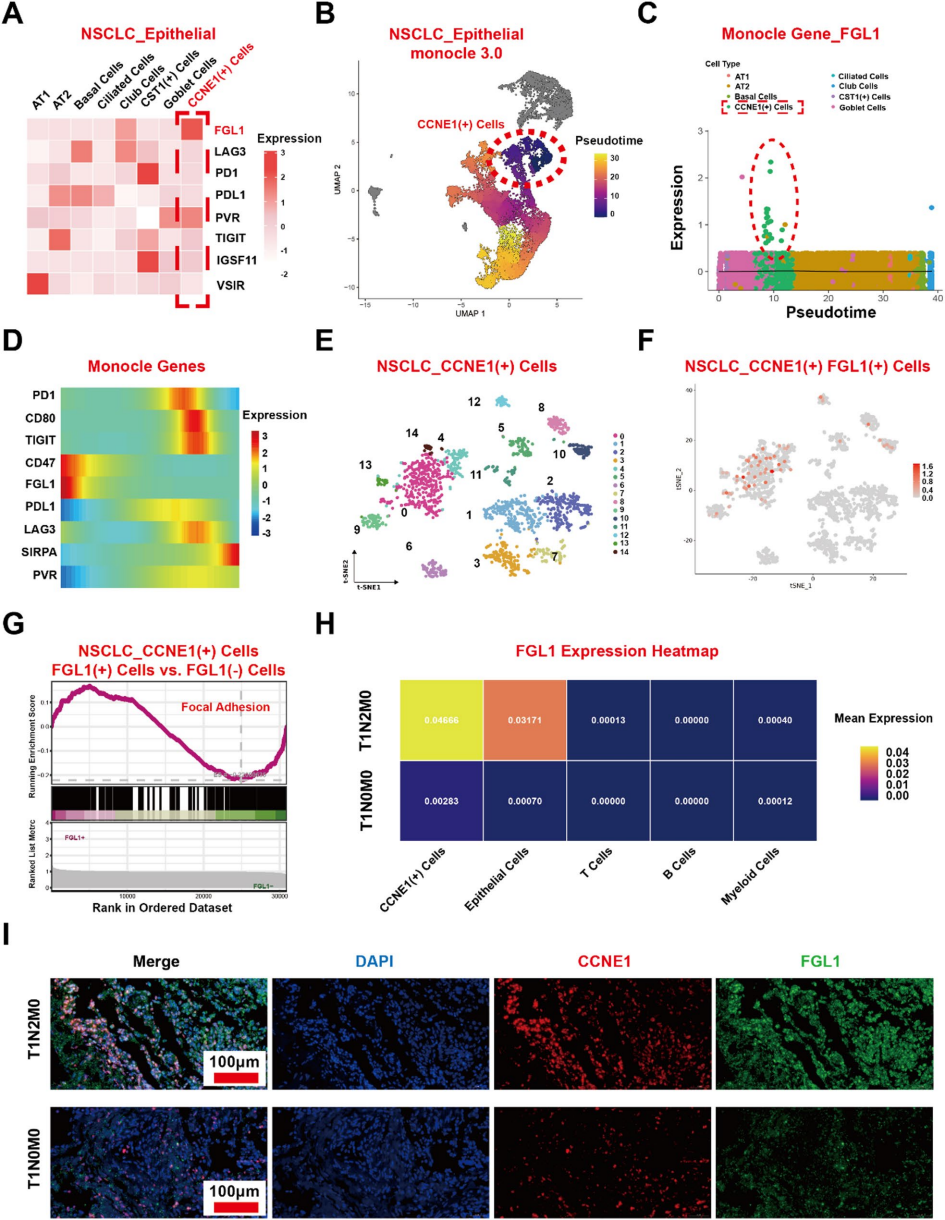
FGL1 is highly expressed in CCNE1(+) cells, and FGL1 appearance coincides with their appearance, indicating its close association with metastasis function. **A** Various immune checkpoint gene expression levels (*FGL1*, *LAG3*, *PD1*, *PD-L1*, *PVR*, *TIGIT*, *IGSF11*, and *VSIR*) are displayed in a heat map of epithelial cells in NSCLC samples. **B** Monocle cell developmental trajectory analysis revealed the differentiation of CCNE1(+) cells. **C**,** D** Expression of FGL1 in the whole trajectory analysis of epithelial cells. **E**,** F** CCNE1(+) cells were divided into FGL1(+) and FGL1(-) cells based on *FGL1* expression. **G** GSEA was used to explore the role of *FGL1* in CCNE1(+) cells based on the differentially expressed genes (DEGs) between FGL1(+) and FGL1(−) cells. **H** FGL1 expression analysis in Epithelial cells (especially in CCNE1(+) cells) and immune cells (T cells, B cells and Myeloid cells). **I** Coimmunofluorescence staining of CCNE1 (red), FGL1 (green) was performed to prove the existence of CCNE1(+) Cells in T1N2M0 (*n* = 15) and T1N0M0 (*n* = 15) NSCLC samples. FGL1, fibrinogen-like protein 1; NSCLC, non-small cell lung cancer; GSEA, Gene Set Enrichment Analysis

*FGL1* is a key gene involved in N2 lymph node metastasis induced by CCNE1(+) Cells. Initially, we believed that CCNE1(+) Cells could transfer from the lung to lymph nodes, which are full of immune cells, without being killed—implying the presence of a specific immune escape ability. Therefore, we selected a group of immune checkpoint genes and analyzed their expressions on CCNE1(+) Cells (*FGL1*, *LAG3*, *PD1*, *PD-L1*, *PVR*, *TIGIT*, *IGSF11*, and *VSIR*), and found that *FGL1* had a specific expression in CCNE1(+) Cells (Fig. 2A). Monocle cell developmental trajectory analysis and differential gene expression comparisons between FGL1(+) and FGL1(-) cells in CCNE1(+) Cells suggested that FGL1 plays an important role in mediating the metastatic function of CCNE1(+) Cells. *FGL1* expression analysis revealed predominant expression in epithelial cells, particularly in CCNE1(+) Cells. Collectively, *FGL1* may have an independent role in promoting tumor metastasis in addition to its currently known immunosuppressive effect.

Monocle cell developmental trajectory analysis revealed that CCNE1(+) Cells were differentiated earliest (Fig. 2B). *FGL1* expression coincided with the appearance of CCNE1(+) Cells and was barely expressed in other cells (Fig. 2C, D). The CCNE1(+) Cells were classified into FGL1(+) and FGL1(-) cells based on *FGL1* expression (Fig. 2E, F). GSEA was used to explore the role of *FGL1* in CCNE1(+) Cells. Differentially expressed genes (DEGs) between FGL1(+) and FGL1(-) cells were mainly enriched in focal adhesion, indicating that *FGL1* promotes metastasis (Fig. 2G). FGL1 expression analysis revealed predominant expression in epithelial cells, particularly in CCNE1(+) cells, compared to immune cells (T, B and myeloid cells), as shown in the heatmap. Besides, FGL1 expression was significantly higher in T1N2M0 samples than in T1N0M0 samples (0.047 vs. 0.002) and no significant difference in immune cell analysis was found between the N0 and N2 groups (Fig. 2H). Collectively, these findings suggest that the non-immune function of CCNE1(+) epithelial cells plays a major role. The existence of CCNE1(+) cells was demonstrated using tissue chip IF staining, and CCNE1 content and *FGL1* expression in T1N2M0 samples (*n* = 15) were significantly higher than those in T1N0M0 samples (*n* = 15) (Fig. 2I). Furthermore, the data indicate that *FGL1*, beyond its known immune regulatory function, also acts as a key functional molecule within CCNE1(+) cells, promoting metastatic regulation. We also verified that *FGL1* is highly expressed in CCNE1(+) cells in these nine lung cancer samples using a public database, which could further support our findings (Supplementary Fig. 3G).

### FGL1 is highly expressed in NSCLC and transcriptionally regulated by ETS1

**Fig. 3:**
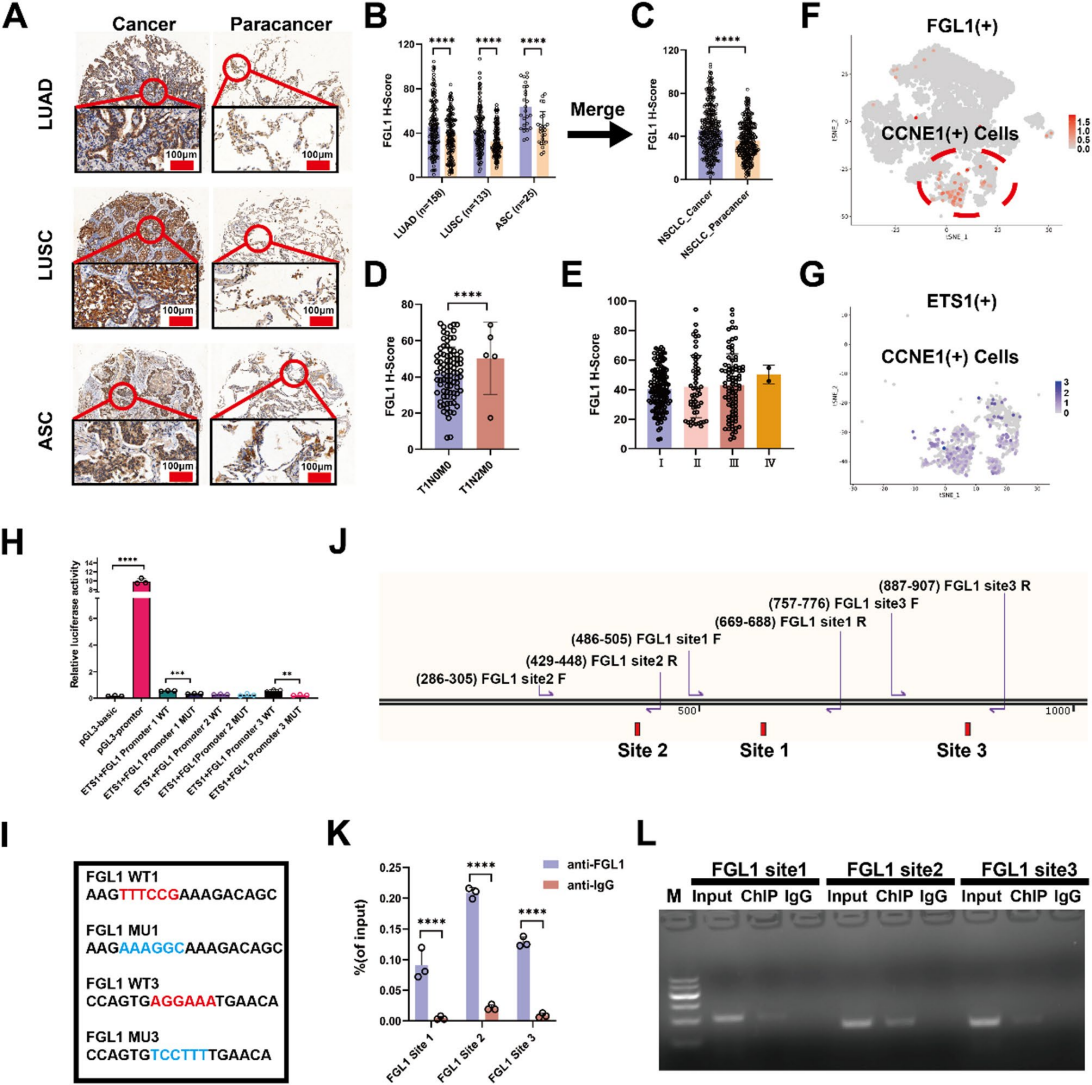
FGL1 is highly expressed in NSCLC and transcriptionally regulated by ETS1. **A–C** Tissue chips were prepared from 316 NSCLC samples, including 158 lung adenocarcinoma, 133 lung squamous cell carcinoma (LUSC), and 25 adenosquamous carcinoma samples, and FGL1 staining was performed using IHC. **D**,** E** FGL1 levels were quantified and compared at all stages of NSCLC. **F**,** G** Single-cell sequencing transcription factor analysis was performed to elucidate the reasons for the high *FGL1* expression in cancer tissues. **H–L** ChIP-qPCR and dual-luciferase reporter gene assays were performed to identify ETS1 and its binding sites on FGL1. In **B**, **C**, **D**, and **E**, data are represented as mean ± SEM, and the Wilcoxon rank test was used for statistical analysis. In **H**, data are represented as mean ± SEM and an unpaired t-test was used for statistical analysis. **P < 0.01; ***P < 0.001; ****P < 0.0001. FGL1, fibrinogen-like protein 1; NSCLC, non-small cell lung cancer; IHC, immunohistochemistry; ETS1, E26 transformation-specific transcription factor 1; ChIP-qPCR, chromatin immunoprecipitation quantitative PCR; SEM, standard error of the mean

IHC confirmed the high expression of *FGL1* in cancer tissues, whereas ChIP-qPCR and dual-luciferase reporter gene assays identified ETS1 as a transcription factor that mediates its high expression. Tissue chips were prepared from 316 NSCLC samples, including 158 lung adenocarcinoma, 133 LUSC, and 25 adenosquamous carcinoma samples. The clinical characteristics detailed in Supplementary Fig. 4A. FGL1 staining and H-score quantification indicated that FGL1 expression was higher in cancer tissues than in paracancerous tissues (*P* < 0.0001; Fig. 3A–C). Notably, T1N2M0 samples exhibited higher FGL1 levels than T1N0M0 samples (*P* < 0.0001; Fig. 3D), while FGL1 expression showed a non-significant increasing trend across advancing disease stages (Fig. 3E). Furthermore, Kaplan-Meier survival analysis revealed no significant association between FGL1 expression and overall prognosis (*P* = 0.492, Supplementary Fig. 4B).

The PROMOT and hTFtargert databases predicted ETS1 to be a transcription factor (Supplementary Fig. 2H). Single-cell sequencing transcription factor analysis was performed to elucidate the reasons for the high *FGL1* expression in cancer tissues. *FGL1* and transcription factor *ETS1* were highly expressed in CCNE1(+) Cells, and ETS1 was initially identified as the transcription factor of *FGL1* (Fig. 3F, G). ChIP-qPCR and dual-luciferase reporter gene assays were performed to identify ETS1 and its binding sites on FGL1. ETS1 was confirmed to be a positive transcriptional regulator of FGL1, with two binding sites (Fig. 3H–L).

### FGL1 regulates glycolysis via the PI3K/AKT/HIF-1α pathway, affecting tumor metastasis

**Fig. 4:**
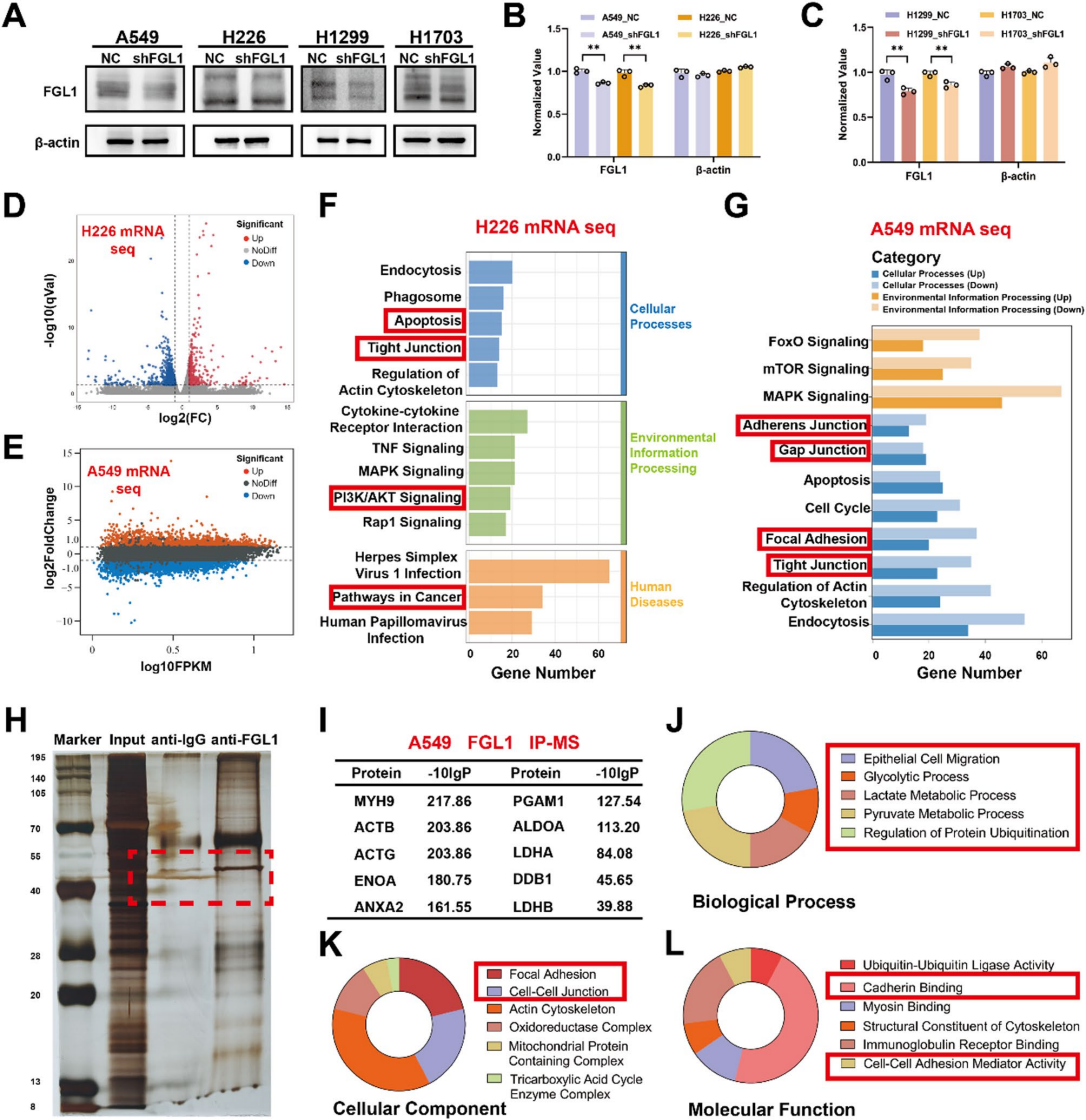
Mass spectrometry and transcriptomic suggest that FGL1 regulates tumor proliferation and metastasis via the PI3K/AKT pathway. **A–C** FGL1 knockdown in A549, H226, H1299, and H1703 cells was achieved using a stable lentivirus, as confirmed by western blotting analysis. **D** Volcano plot showing the DEGs after FGL1 knockdown in H226 cells by transcriptome sequencing. **E** Volcano map showing the DEGs in A549 cells after FGL1 knockdown. **F** KEGG analysis showed the enrichment of DEGs based on transcriptome sequencing of H226 cells. **G** KEGG differential gene analysis showed that the DEGs were enriched in tumor progression-related pathways of A549 cells. **H–L** CO-IP and mass spectrometry were performed to identify proteins closely associated with FGL1 in A549 cells. In **B** and **C**, data are represented as mean ± SEM and an unpaired t-test was used for statistical analysis. **P < 0.01. FGL1, fibrinogen-like protein 1; PI3K, phosphoinositide 3-kinase; DEGs, differentially expressed genes; KEGG, Kyoto Encyclopedia of Genes and Genomes; Co-IP, co-immunoprecipitation; SEM, standard error of the mean

FGL1 plays a regulatory role in the malignant progression of tumors via the PI3K/AKT pathway. FGL1 knockdown in A549, H226, H1299, and H1703 cells was achieved using a stable lentivirus, as confirmed by western blotting (*P* < 0.01; Fig. 4A–C). Transcriptome sequencing in H226 cells revealed 322 upregulated and 1,176 downregulated genes after FGL1 knockdown, as shown in the volcano plot (Fig. 4D). KEGG analysis further showed enrichment in pathways related to tumor proliferation and migration, including Apoptosis, Tight Junction, PI3K/AKT Signaling Pathway, among other cancer pathways (Fig. 4F). Similarly, transcriptome sequencing of A549 cells revealed the upregulation of 2,103 genes and downregulation of 3,187 genes after *FGL1* knockdown (Fig. 4E). KEGG analysis showed that DEGs were mainly enriched in signaling pathways related to tumor proliferation and migration, including Adherens junction, Gap junction, Focal adhesion, and Tight junction (Fig. 4G).

Co-immunoprecipitation (Co-IP) was performed to identify proteins closely associated with FGL1. Mass spectrometry and Co-IP in A549 cells (Fig. 4H, I) revealed that FGL1-related proteins were also enriched in functions regulating tumor migration such as Focal Adhesion and Cell-Cell Junction. Notably, the results in A549 cells indicated *FGL1* involvement in glycolysis, lactate metabolism, and pyruvate metabolism, through the regulation of significant glycolysis molecules including ENOA, PGAM1, LDHA, and LDHB (Fig. 4J–L).

**Fig. 5:**
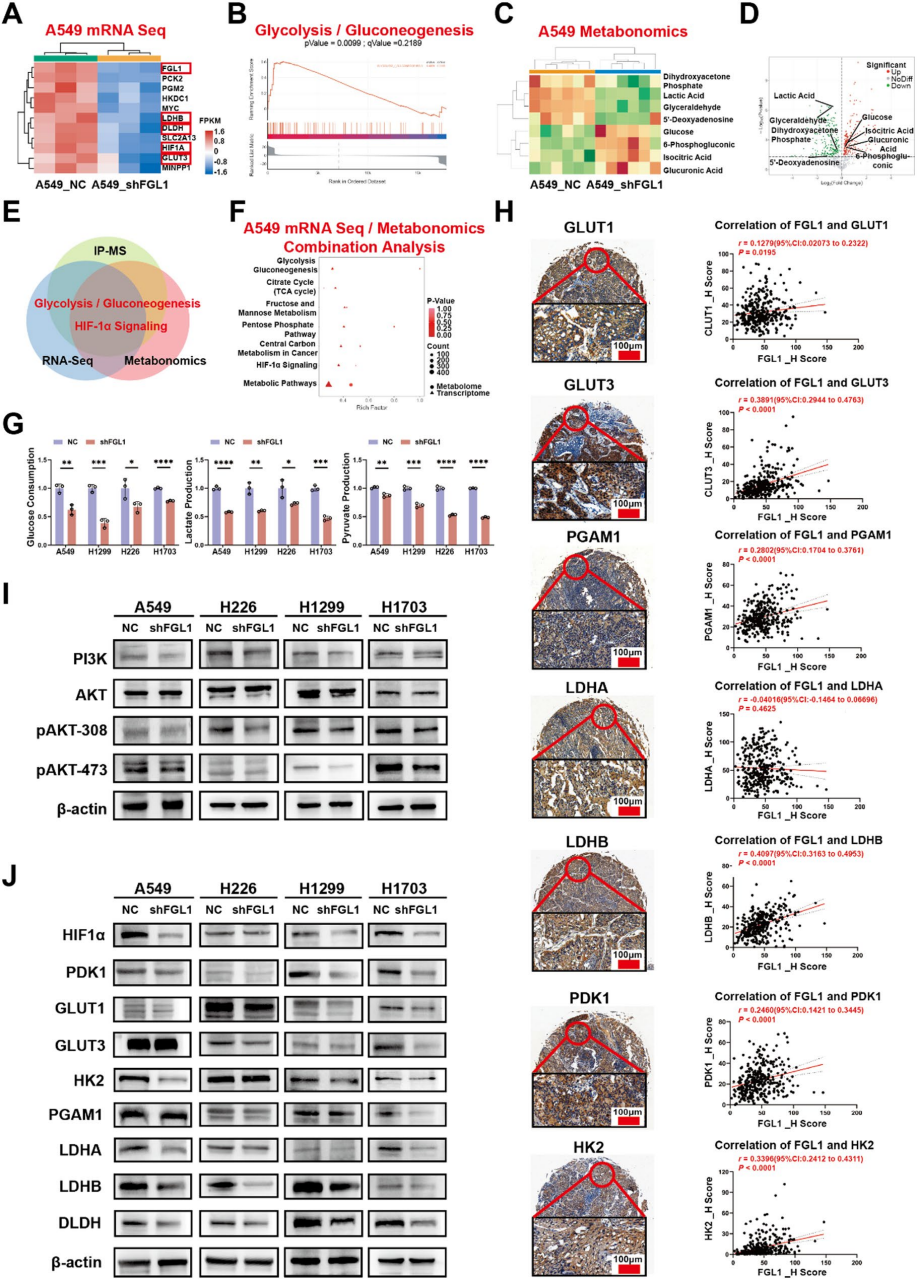
Mass spectrometry, transcriptomic, and metabonomic analyses suggest that FGL1 regulates glycolysis via the HIF-1α pathway. **A**,** B** GSEA of transcriptome sequencing revealed that DEGs between A549_NC and A549_shFGL1 cells were enriched in glycolysis. **C**,** D** Metabonomics showed decreased levels of important glycolytic metabolites after FGL1 knockdown. **E**,** F** Combined analysis of mass spectrometry, RNA-seq, and metabonomics indicated that FGL1 knockdown affected glycolysis and the HIF-1α signaling pathway. **G** Glucose consumption, lactate production, and pyruvate production assays were performed in the four NSCLC cell lines to evaluate the effect of FGL1 on tumor glycolysis. **H** IHC and H-score quantification in NSCLC tissue chips showed a correlation between FGL1 and glycolysis molecules. **I** Western blotting confirmed that *FGL1* knockdown inhibited the PI3K/AKT signaling pathway. **J** Western blotting confirmed that *FGL1* knockdown inhibited the HIF-1α signaling pathway and glycolysis. In **G**, data are represented as mean ± SEM and an unpaired t-test was used for statistical analysis. *P < 0.05; **P < 0.01; ***P < 0.001; ****P < 0.0001. FGL1, fibrinogen-like protein 1; HIF-1α, hypoxia-inducible factor 1-alpha; GSEA, Gene Set Enrichment Analysis; DEGs, differentially expressed genes; NC, negative control; scRNA-seq, single-cell RNA sequencing; IHC, immunohistochemistry; SEM, standard error of the mean; PI3K, phosphoinositide 3-kinase

FGL1 plays a regulatory role in tumor glycolysis through the PI3K/AKT/HIF-1α pathway. Transcriptome sequencing and metabonomics were performed to further verify the involvement of FGL1 in glycolysis, as indicated by mass spectrometry. GSEA of transcriptome sequencing revealed that DEGs between A549_NC and A549_shFGL1 were enriched in glycolysis; the expression of key molecules in glycolysis was significantly decreased after *FGL1* knockdown, including LDHB, DLDH, HIF-1α, and GLUT3 (Fig. 5A, B). Metabonomics showed decreased levels of important glycolytic metabolites, Such as dihydroxyacetone phosphate, lactic acid, and glyceraldehyde, alongside increased levels of other glycolytic metabolites Such as glucose. These were related to the changes in other glucose metabolism molecules such as 5′-deoxyadenosine, 6′-phosphogluconic, isocitric acid, and glucuronic acid, after *FGL1* knockdown (Fig. 5C, D). Combined analysis of mass spectrometry, RNA-seq, and metabonomics indicated that FGL1 knockdown affected glycolysis and the HIF-1α signaling pathway (Fig. 5E, F). Further, glucose consumption, lactate production, and pyruvate production assays in the four NSCLC cell lines to evaluate the effect of FGL1 on tumor glycolysis revealed significant inhibition of glycolysis after *FGL1* knockdown (*P* < 0.05; Fig. 5G). Besides, IHC and H-score quantification in the NSCLC tissue chips showed positive correlations between FGL1 and glycolysis markers. FGL1 was positively correlated with GLUT3 (*r =* 0.3891, *P* < 0.0001), GLUT1 (*r =* 0.1279, *P* = 0.0195), LDHB (*r =* 0.4097, *P* < 0.0001), PGAM1 (*r =* 0.2802, *P* < 0.0001), HK2 (*r =* 0.3396, *P* < 0.0001), and PDK1 (*r =* 0.2460, *P* < 0.0001) (Fig. 5H). Western blotting confirmed that *FGL1* knockdown inhibited the expression of some molecules in the PI3K/AKT signaling pathway such as PI3K, pAKT-308, and pAKT-473 in the PI3K signaling pathway (Fig. 5I). Some protein changes were verified to explore the effect of FGL1 on the HIF-1α signaling pathway and glycolysis. The expression of key molecules involved in glycolysis, including HIF-1α, PDK1, GLUT1, LDHA, LDHB, and DLDH, decreased after *FGL1* knockdown (Fig. 5J).

**Fig. 6:**
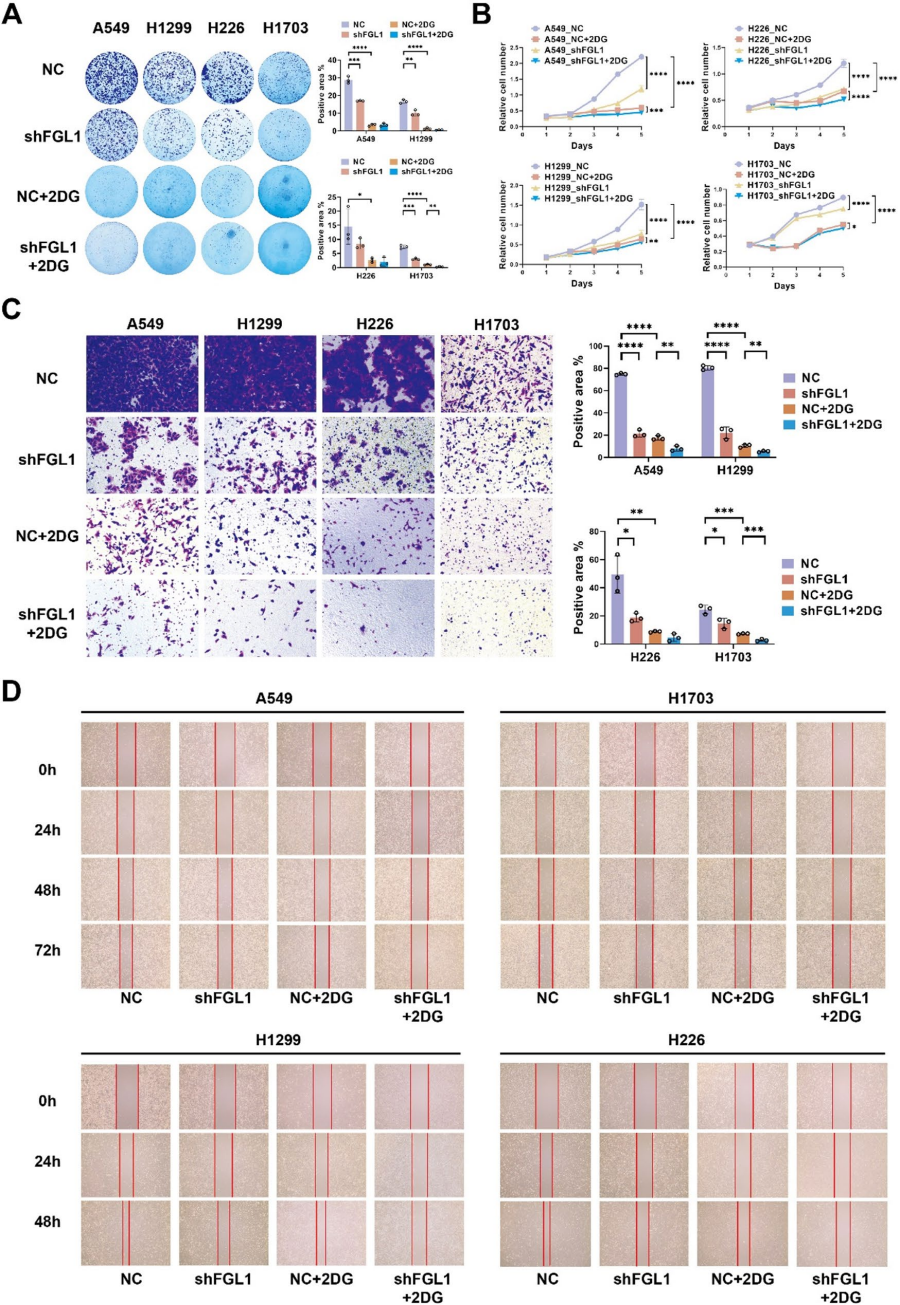
FGL1 knockdown inhibited tumor progression, including proliferation and metastasis, by affecting the glycolytic pathway. **A**,** B** Colony formation and CCK-8 assays were used to explore the proliferation of NC (negative control) and FGL1 KD (knockdown) A549, H226, H1299, and H1703 cells in regular and glycolytic inhibition environments (2-deoxy-D-glucose, 9 mMol/mL). **C**,** D** Transwell and scratch tests were used to explore the metastasis of these four cell lines in regular and glycolytic-inhibition environments. In **A**, **B**, and **C**, data are represented as mean ± SEM and an unpaired t-test was used for statistical analysis. *P < 0.05; **P < 0.01; ***P < 0.001; ****P < 0.0001. FGL1, fibrinogen-like protein 1; KD, knockdown; NC, negative control; CCK-8, Cell Counting Kit-8; SEM, standard error of the mean

**Fig. 7:**
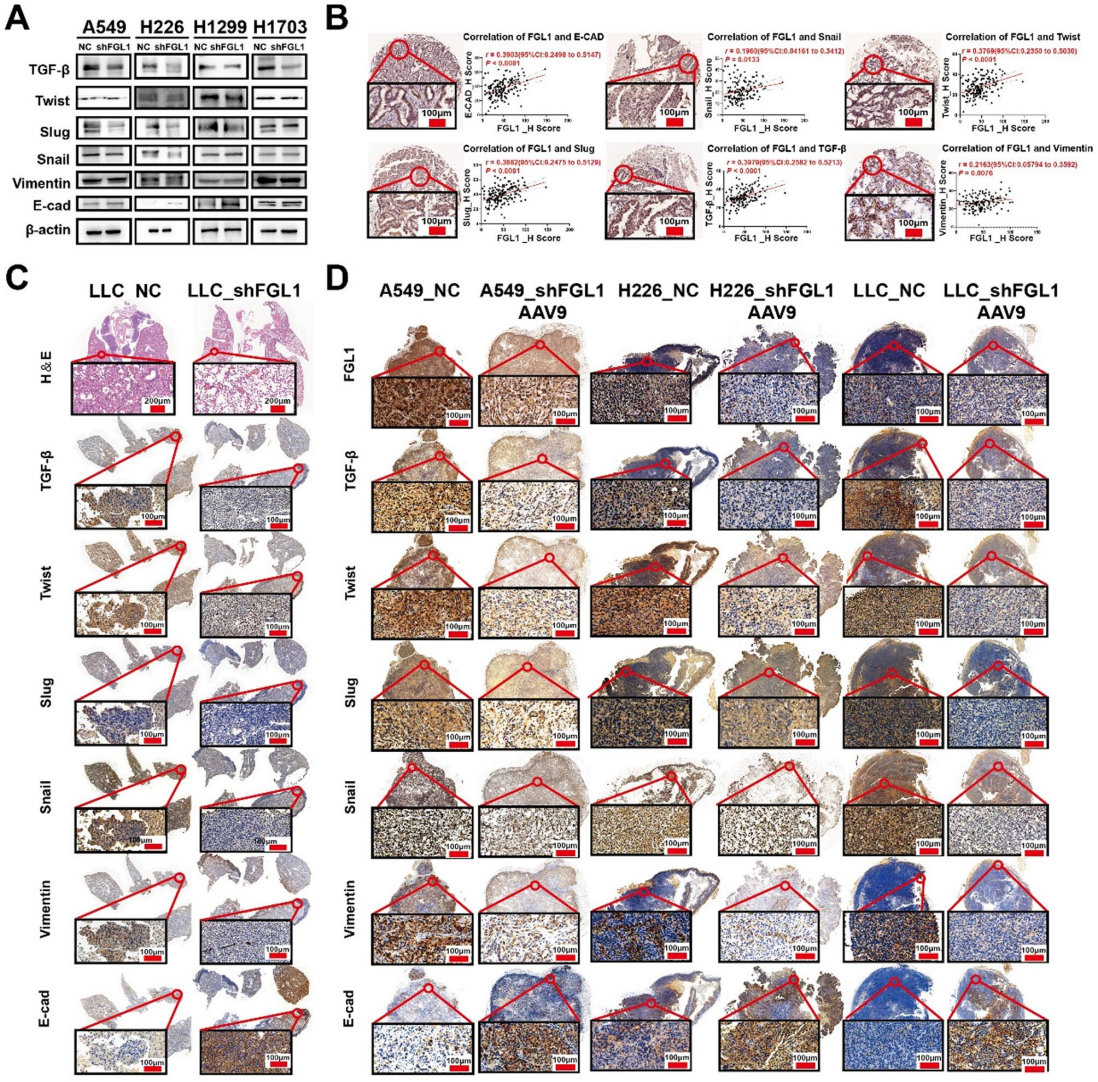
The expression of key EMT molecules was inhibited after FGL1 knockdown. **A** Western blotting confirmed that *FGL1* downregulation significantly inhibited the expression of EMT-related genes. **B** IHC and H-score quantification in LUSC tissue chips showed a correlation between FGL1 and EMT markers (*n* = 133). **C** FGL1 knockdown in LLC cell lines significantly inhibited tumor metastasis in pulmonary tumors in situ in nude mice (Fig. 8C). IHC was performed to verify the changes in key EMT molecules after *FGL1* knockdown. **D** shFGL1_ AAV9 was constructed for intratumoral injection to achieve FGL1 knockdown in LLC, A549, and H226 cell lines. IHC was performed to verify the changes in EMT-related molecules after shFGL1_ AAV9 administration. FGL1, fibrinogen-like protein 1; EMT, epithelial-mesenchymal transition; IHC, immunohistochemistry; LUSC, lung squamous cell carcinoma; LLC, Lewis lung carcinoma; AAV9, Adeno-Associated Virus serotype 9

FGL1 knockdown inhibited tumor progression, including proliferation and metastasis by affecting glycolysis. In vitro characterization experiments were performed to further investigate the function of FGL1 on Tumor proliferation, metastasis, and glycolysis. A glycolytic inhibitory environment was created using 2-DG at 9 mMol/mL. Colony formation and CCK-8 assays were used to explore the proliferation of NC and FGL1_KD A549, H226, H1299, and H1703 cells. The FGL1_KD cells had lower proliferation rates than the NC cells. In the glycolytic inhibition environment, the inhibitory effect of *FGL1* knockdown on proliferation was significantly reduced compared with the normal environment (*P* < 0.05; Fig. 6A, B). Transwell and scratch tests indicated that FGL1 KD cells had reduced migration rates compared to the control group, and this effect was also significantly inhibited in the glycolytic inhibition environment (*P* < 0.05; Fig. 6C, D). Importantly, *FGL1* downregulation significantly inhibited the expression of EMT-related molecules such as TGF-β, Slug, and Snail, while increasing E-cadherin expression, which is important for tumor metastasis (*P* < 0.05; Fig. 7A). IHC and H-score quantification in LUSC tissue chips showed positive correlations between FGL1 and EMT process markers. FGL1 was positively correlated with E-cadherin (*r* = 0.3903, *P* < 0.0001), Snail (*r* = 0.1960, *P* = 0.0133), Twist (*r* = 0.3769, *P* < 0.0001), Slug (*r* = 0.3882, *P* < 0.0001), TGF-β (*r* = 0.3979, *P* < 0.0001), and Vimentin (*r* = 0.2136, *P* = 0.0076) (Fig. 7B). These findings suggests that FGL1 regulates tumor proliferation and metastasis by regulating glycolysis.

### FGL1 knockdown inhibited tumor metastasis, especially for lymph node metastasis by inhibiting lymph tube formation

As mentioned above, FGL1 acts as an immune checkpoint in NSCLC. Node and NCG mice were used as experimental animal models [25–27] to investigate the independent regulation of proliferation and metastasis by the FGL1 protein.

**Fig. 8:**
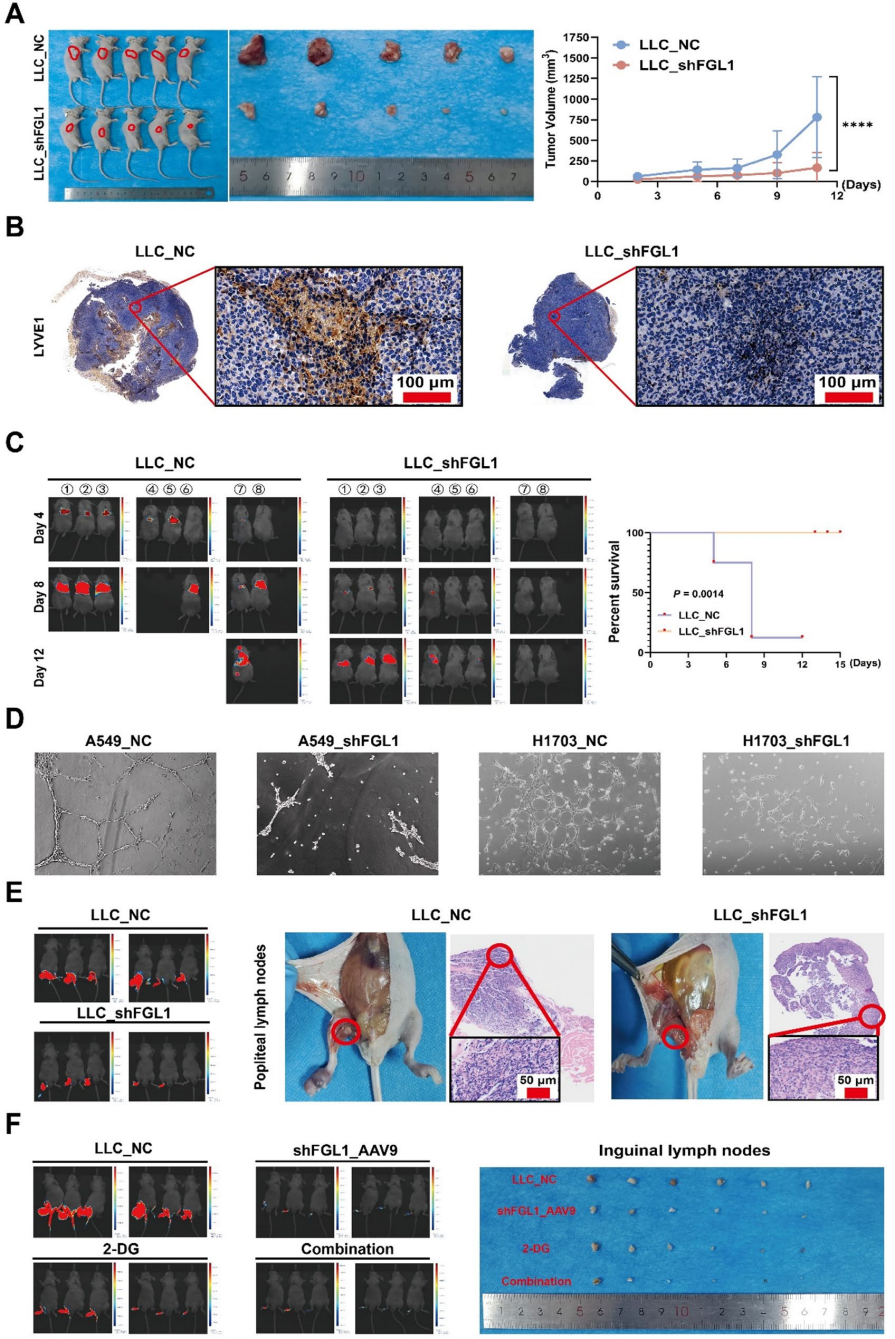
*FGL1* knockdown inhibited tumor proliferation, metastasis, and lymph tube formation to inhibit lymph node metastasis. **A** Subcutaneous tumor-bearing nude mice showed that *FGL1* knockdown in LLC cell lines significantly inhibited tumor proliferation (*n* = 10). **B** Staining for LYVE1 was performed in subcutaneous tumors derived from LLC_NC and LLC_shFGL1 cells. **C** LLC_NC and LLC_shFGL1 cells were injected into the left lung of NCG mice in situ, and Tumor formation and metastasis were observed via small animal imaging at 4, 8, and 12 days. Survival analysis was performed (*n* = 16). **D** A549 and H1703 cells with and without FGL1 knockdown were co-cultured with lymphatic endothelial cells, and lymph tube formation was observed. **E** Footpad lymph node metastasis test was performed, and LLC_NC and LLC_shFGL1 cells were injected into the footpads. Metastasis was observed in the inguinal and popliteal lymph nodes. **F** shFGL1_AAV9 and 2DG combination therapy was performed after tumor formation in the footpad, and a therapeutic effect was observed. In **A** and **C**, data are represented as mean ± SEM and an unpaired t-test was used for statistical analysis. ****P < 0.0001. FGL1, fibrinogen-like protein 1; LLC, Lewis lung carcinoma; NC, negative control; LYVE-1, lymphatic vessel endothelial receptor 1; 2-DG, 2-deoxy-D-glucose; SEM, standard error of the mean

Downregulation of FGL1 inhibited tumor proliferation in vivo. Stable Lewis lung carcinoma (LLC) and H226 cell lines with low *FGL1* knockdown were constructed using lentivirus vectors. Subcutaneous tumor-bearing nude mice showed that *FGL1* knockdown in LLC and H226 cell lines significantly inhibited tumor proliferation **(***P* < 0.05; Fig. 8A and Supplementary Fig. 6A). Glycolytic molecule staining in the LLC_NC and LLC_shFGL1 groups revealed significant inhibition of the expression of key glycolysis molecules after *FGL1* knockdown, including HK2, GLUT1, GLUT3, LDHA, LDHB, PDK1, and PGAM1 (Supplementary Fig. 5).

*FGL1* downregulation inhibited tumor metastasis in vivo. An in situ NCG mouse lung tumor model was used to simulate lung tumor metastasis to other sites. LLC_NC and LLC_shFGL1 cells were injected into the left lung of NCG mice in situ. Next, Tumor formation and metastasis were observed using small animal imaging at 4, 8, and 12 days. Longitudinal imaging revealed that 80% of control mice developed pulmonary metastases by day 8, with rapid progression leading to mortality. One control mouse exhibited distal metastases by day 12 (Fig. 8C). HE staining of lungs from terminal control mice showed significant tumor cell infiltration and alveolar wall thickening, confirming widespread intrapulmonary metastasis (Fig. 7C), consistent with metastatic burden-induced death. Conversely, FGL1_KD mice showed minimal metastatic lesions and significantly prolonged survival (*P* = 0.0014). The expression of EMT-related molecules decreased after *FGL1* knockdown, with E-cadherin expression increasing (Fig. 7C).

Downregulation of *FGL1* inhibited lymph tube formation and, ultimately, tumor lymph node metastasis. A549 cells with and without *FGL1* knockdown were co-cultured with human lymphatic ECs, and lymphatic tube formation was observed. Lymph tube formation was significantly inhibited in the *FGL1* knockdown group (Fig. 8D). Staining of lymphatic vessel endothelial receptor-1 (LYVE-1) was performed in the subcutaneous tumor derived from LLC_NC and LLC_shFGL1. The expression of LYVE-1 was significantly inhibited in the LLC_shFGL1 group, proving that *FGL1* knockdown inhibited lymph tube formation (Fig. 8B). A footpad lymph node metastasis test was performed, and LLC_NC and LLC_shFGL1 cells were injected into the footpad. The metastasis of inguinal and popliteal lymph nodes was observed, and lymph node metastasis was found to be significantly inhibited in the *FGL1* knockdown group (*P* < 0.05; Fig. 8E).

### Anti-FGL1 treatment is a promising strategy for inhibiting NSCLC metastasis

To further explore the possibility of clinical transformation, adeno-associated viruses were used to investigate the efficacy of *FGL1* knockdown in vivo on tumor proliferation and metastasis, particularly lymph node metastasis. LLC_NC cells were injected into the footpad to perform lymph node metastasis tests. Additionally, shFGL1_AAV9 was injected into the footpad after Tumor formation, 2-DG was injected intraperitoneally, and combination therapy was performed. The results showed that shFGL1_AAV9 administration effectively inhibited lymph node metastasis (Fig. 8F).

shFGL1_AAV9 was constructed for *FGL1* knockdown to inhibit tumor proliferation in vivo. A549, H226, and LLC cell lines were used for subcutaneous tumor-bearing nude mice, and shFGL1_AAV9 was used for intratumoral injection when the Tumor volume reached 150–200 mm^3^ to achieve *FGL1* knockdown in vivo. All three cell lines showed that intratumoral inhibition of *FGL1* significantly inhibited tumor proliferation (*P* < 0.05; Supplementary Fig. 6B–E). Staining for EMT-related molecules in all three groups indicated that *FGL1* knockdown significantly induced the expression of E-cadherin and decreased the expression of TGF-β, Twist, Slug, Snail, and Vimentin (Fig. 7D). Thus, *FGL1* knockdown in vivo inhibited tumor proliferation by suppressing glycolysis.

Furthermore, shFGL1_AAV6 was constructed for *FGL1* knockdown to inhibit lung tumor metastasis in vivo. We injected LLC intrapulmonary, followed by shFGL1_AAV6 administration via a non-invasive tracheal injection after 2 days, to verify the therapeutic effect of shFGL1_AAV6. The therapeutic effect of shFGL1_AAV6 was observed using small animal imaging at 4, 8, and 12 days. Notably, *FGL1* knockdown in situ significantly inhibited tumor metastasis and improved survival rates (*P* = 0.0751; Supplementary Fig. 6F).

## Discussion

In conclusion, our study identified a cluster of cells, named CCNE1(+) Cells, with a high degree of malignancy and high expression of FGL1, which were more prevalent in T1N2M0 than in T1N0M0 samples. The migration promoting function of FGL1(+) cells in this group may mediate lymph node metastasis in stage T1 NSCLC, particularly N2 lymph node metastasis, and FGL1 expression coincides with the appearance of this cell group. Differential gene analysis indicated that FGL1 itself may promote metastasis in addition to its classical immune regulatory function as an immune checkpoint, suggesting that FGL1 may be closely related to lymph node metastasis in this cell group. Here, we proposed the “shield machine cutter” role of FGL1 in mediating N2 lymph node metastasis in T1 NSCLC, which, like opening up a subway channel, opens a lymph node metastasis channel. Furthermore, ETS1 was identified as the transcription factor responsible for high *FGL1* expression in NSCLC. Mass spectrometry combined with transcription sequencing revealed that FGL1 may participate in the EMT process of NSCLC via the PI3K/AKT/HIF-1α pathway, affecting glycolysis molecules such as HIF-1α, PDK1, GLUT1, LDHA, LDHB, and DLDH, as confirmed through in vivo and in vitro experiments. Further analyses suggested that FGL1 promotes tumor proliferation, metastasis and lymph tube formation, and finally induces lymph node metastasis, which is verified in vivo and in vitro, whereas its knockdown inhibits these processes. Finally, the anti-FGL1 therapeutic effects of shFGL1-AAV6 and shFGL1-AAV9 were verified in animal models, and the effect of *FGL1* knockdown on the expression of glycolysis- and EMT-related molecules was verified by IHC. This study proposes a mechanism of FGL1-mediated N2 lymph node metastasis in stage T1 NSCLC and a related therapeutic strategy targeting FGL1.

N2 lymph node metastasis in T1 NSCLC is high, although the underlying mechanism remains unclear. Lung cancer has the highest mortality rate among all Tumors, with NSCLC accounting for approximately 80% of all cases. Patients with stage T1 NSCLC, defined by a lung cancer tumor < 3 cm, are typically considered clinically as candidates for radical surgery and have a cure plan [28]. However, in current clinical diagnosis and treatment, lymph node metastasis is found in approximately 30% of patients with stage T1 NSCLC, and the higher the intracranial solid proportion, the greater the probability of metastasis [3]. Even in clinically operable cT1N0M0 stage lung cancer, 20–30% of patients have N1–N2 lymph node metastasis pathologically confirmed after surgery (so called “occult nodal metastasis”). Additionally, approximately 30% of patients with stage IA NSCLC experience recurrence and metastasis after surgery [29], which seriously affects their prognosis. However, the underlying mechanism of lymph node metastasis in T1 NSCLC remains unknown, necessitating further research into potential cell populations or genes involved in the regulation of metastasis. Therefore, active exploration of lymph node metastasis in T1 NSCLC has important clinical significance. Our study identified a group of CCNE1(+) Cells and demonstrated the mechanism by which they mediate metastasis via FGL1.

FGL1 may be associated with immune cell suppression, although evidence of a direct correlation is limited. FGL1, a Ligand of lymphocyte-activation gene 3 (LAG3) [30], also known as hepassocin, HRFREP-1, and FREP1 [31–34], is primarily expressed on the surface and within the cytoplasm of lung and breast cancer cells. FGL1 can negatively regulate the tumor immune microenvironment by inhibiting T cell activation through binding to LAG3 [31–34]. Currently, the predominant view is that the FGL1 receptor LAG3 directly participates in immune cell inhibition, and LAG3 overexpression may further enhance FGL1-mediated T cell inhibition [30]. Liu et al. found that LAG3-related genes are mainly enriched in PD-1/PD-L1 related pathways, cytotoxicity, antigen presentation, and other related pathways, with high expression of LAG3 correlating with decreased T and B cell numbers [35]. In breast cancer, Du et al. believe that the co-expression of LAG3 and PD1 is closely related to the inhibition of T cell secretion function [36, 37], while others suggest that the high expression of LAG3 is closely related to the increase in NK and DC cells in breast cancer, lung adenocarcinoma, and LUSC [38]. In 3A9 T cells with LAG3 overexpression, FGL1 significantly inhibits T cell proliferation, and anti-FGL1 monoclonal antibody can lead to increased TNF-α and IFN-γ levels, thereby restoring T cell function [30]. Our previous study demonstrated that FGL1 downregulation in lung adenocarcinoma prevents Jurkat T cell apoptosis, and accelerates TNF-α secretion [22], suggesting an FGL1-related immune mechanism as a potential cause of N2 lymph node metastasis in T1 NSCLC via FGL1-mediated immune escape of CCNE1(+) Cells.

FGL1 may promote tumor growth, invasion, and migration; however, its role in lung cancer requires further investigation. FGL1 may be present as an oncogene in colon cancer, in FGL1-knockout mouse models implanted with MC38 colon cancer cells, the growth rate of colon cancer cells was significantly reduced compared to the control group, and the application of anti-FGL1 monoclonal antibodies showed favorable antitumor effects [30]. Conversely, *FGL1* may act as a tumor suppressor gene in lung adenocarcinoma with LKB1 mutation, the SGC-7901 gastric cancer cell line, and liver cancer. In A549 lung adenocarcinoma cells with LKB1 overexpression, *FGL1* knockdown significantly increases growth rate, migration of lung cancer cells, angiogenesis, and EMT, potentially mediating lung cancer metastasis [39]. In SGC-7901 cells, *FGL1* knockdown significantly promotes the invasion, proliferation, and migration of tumor cells [40]. Similarly, in liver cancer, *FGL1* expression decreases with disease progression, and compared with wild-type mice, the growth rate of liver cancer in *FGL1*-knockout mice is faster, possibly involving the AKT and mTOR pathways [41]. Therefore, the role of *FGL1* in different tumors varies, acting as a tumor suppressor gene in lung adenocarcinoma with LKB1 mutation. However, few similar studies have been reported, and the role of *FGL1* in other types of lung adenocarcinoma needs to be further studied and verified. Our previous research demonstrated the proliferation and immune regulation of FGL1 in tumors, which indicated that *FGL1* downregulation inhibits lung adenocarcinoma cell proliferation via MYC signaling [21]. In this study, we demonstrated an independent metastasis regulation mechanism of FGL1 via the PI3K/AKT/HIF-1α signaling pathway, in addition to immune regulation.

We propose, for the first time, that FGL1 plays a role in glycolytic regulation. Some other immune checkpoints play a role in glycolytic regulation, such as PD-1/PD-L1, which is involved in immune regulation related to glucose metabolism. Previous studies have shown that when PD-1 binds to PD-L1, it transmits a signal via the T cell receptor (TCR). Src family kinases (such as lymphocyte-specific protein tyrosine kinase (Lck) and Fyn) phosphorylate tyrosine at the immunoreceptor tyrosine inhibitory motif and immunoreceptor tyrosine switching motif sites on the PD-1 cytoplasmic tail, thereby recruiting phosphatase SHP-2. The downregulated Ras/AKT pathway is involved in TCRs and the PI3K/AKT/mTOR signaling pathway is involved in CD28 co-stimulatory receptors, which are key regulatory factors of T cell metabolism. Integration of TCR signals, CD28 co-stimulation, and cytokine receptors promotes glycolysis, and the upregulation of PD-1 counteracts these pathways, reducing T cell glycolysis, proliferation, and activation [42–44]. CTLA4 binds to protein B7, inhibiting CD28 signal transduction and protein phosphatase 2a, which suppresses AKT/mTORC1 activity in the PI3K/AKT/mTORC1 signal transduction pathway and downregulates key enzymes in T cell metabolism, including GLUT1, Hexokinase 2, and pyruvate kinase isoenzyme M2, preventing activation-induced glycolysis. This reduces the differentiation of activated T cells into effector cells [45, 46]. He et al. promoted the interaction between TIGIT and CD155 through co-culture of CD8^+^ T cells with gastric cancer cells, and found that gastric cancer cells deprived CD8 T cells of glucose, decreasing their efficacy. Blocking TIGIT on CD8^+^ T cells increased AKT/mTOR pathway phosphorylation, thereby increasing their metabolism and cytokine production. In addition, blocking CD155 on gastric cancer cells effectively controlled tumor growth, reduced tumor glucose metabolism, and enhanced the metabolism of nutritionally competitive CD8^+^ T cells and the immune response [47]. In triple-negative breast cancer, the TIGIT/CD155 pathway was also found to inhibit PI3K, p-AKT, and p-mTOR expression, reducing glucose uptake and lactic acid production in CD8^+^ T cells, leading to CD8^+^ T cell activation and impairment of effector functions in the tumor microenvironment. Blocking TIGIT/CD155 effectively reversed this effect [48]. Our mass spectrometry, transcriptomic, and metabonomic analyses suggest that FGL1 regulates glycolysis by mediating the PI3K/AKT/HIF-1α pathway to influence malignant progression such as tumor proliferation and metastasis. We demonstrated that FGL1 affects glycolytic molecules in vivo and in vitro, particularly glucose consumption, lactate production and pyruvate production.

Currently, there are no clinical monoclonal antibodies that target FGL1, necessitating further foundational research. Various monoclonal drugs targeting LAG3, such as IMP321 and relatlimab, have been undergoing clinical trials [49, 50], with trials for monotherapy and combination therapy also being conducted globally [50]. These trials include breast cancer [51], liver cancer [52], prostate cancer [53], colorectal cancer [54], and lymphoma [55]. However, the combination treatment strategy is relatively simple, mostly using anti-PD-1 or anti-PD-L1 monoclonal antibodies [54, 56, 57]. Besides, the dysregulation of epigenetic processes may mediate the changes in the efficacy of immunotherapy for lung cancer, the current clinical trials of epigenetic therapy has been reviewed [58]. FGL1, as a cancer gene, is also an immune checkpoint gene, studies on the epigenetics of FGL1 may better and more comprehensively explore the function of FGL1 in lung cancer, providing a good theoretical basis for further designing targeted drugs for FGL1, the epigenetic changes of FGL1 may be the next therapeutic target for clinical transformation. Due to there are no reports of anti-FGL1 monoclonal antibodies that can be used clinically, likely resulting from insufficient basic research and in vivo trial data, further, to better provide evidence for clinical transformation targeting FGL1, we constructed AAV6-shFGL1 and AAV9-shFGL1 and verified their therapeutic effects in vivo by non-invasive endotracheal injection and intratumoral injection, respectively, potentially identifying a new antitumor therapeutic target.

However, our study has some limitations. First, due to sampling and hardware constraints, we could not extract the CCNE1(+) Cells for research; second, in terms of immune escape from this population of cells, some other immune checkpoints showed higher expression in the CCNE1(+) Cells in lymph nodes, which may influence future tumor progression; additionally, marker genes with higher tumor intrinsic expression in CCNE1(+) Cells may also promote metastasis, necessitating further verification.

Overall, our research focused on the role of FGL1 in NSCLC and the mechanisms of its involvement in lymph node metastasis in stage T1 NSCLC, with the aim of developing a feasible treatment strategy targeting FGL1, supported by preliminary preclinical verification to provide evidence for identifying new immunotherapy targets.

## Supplementary Information

Supplementary Material 1.

## Data Availability

The entire original code has been deposited at Figshare and is publicly available as of the date of publication. DOIs are listed below: 10.6084/m9.figshare.28625303; Sequence data that support the findings of this study have been deposited in the GEO dabatase as GSE 293360.
