# Supplementary material for: FGL1-mediated lymph node metastasis in stage T1 non-small cell lung cancer: therapeutic targeting
Source: Exp Hematol Oncol. 2025 Sep 29;14:117. doi: 10.1186/s40164-025-00709-5 (PMC12481761; doi:10.1186/s40164-025-00709-5)
Supplement: Supplementary file 1 — Supplementary Material 1. [file 40164_2025_709_MOESM1_ESM.docx]

**Supplementary Figures**


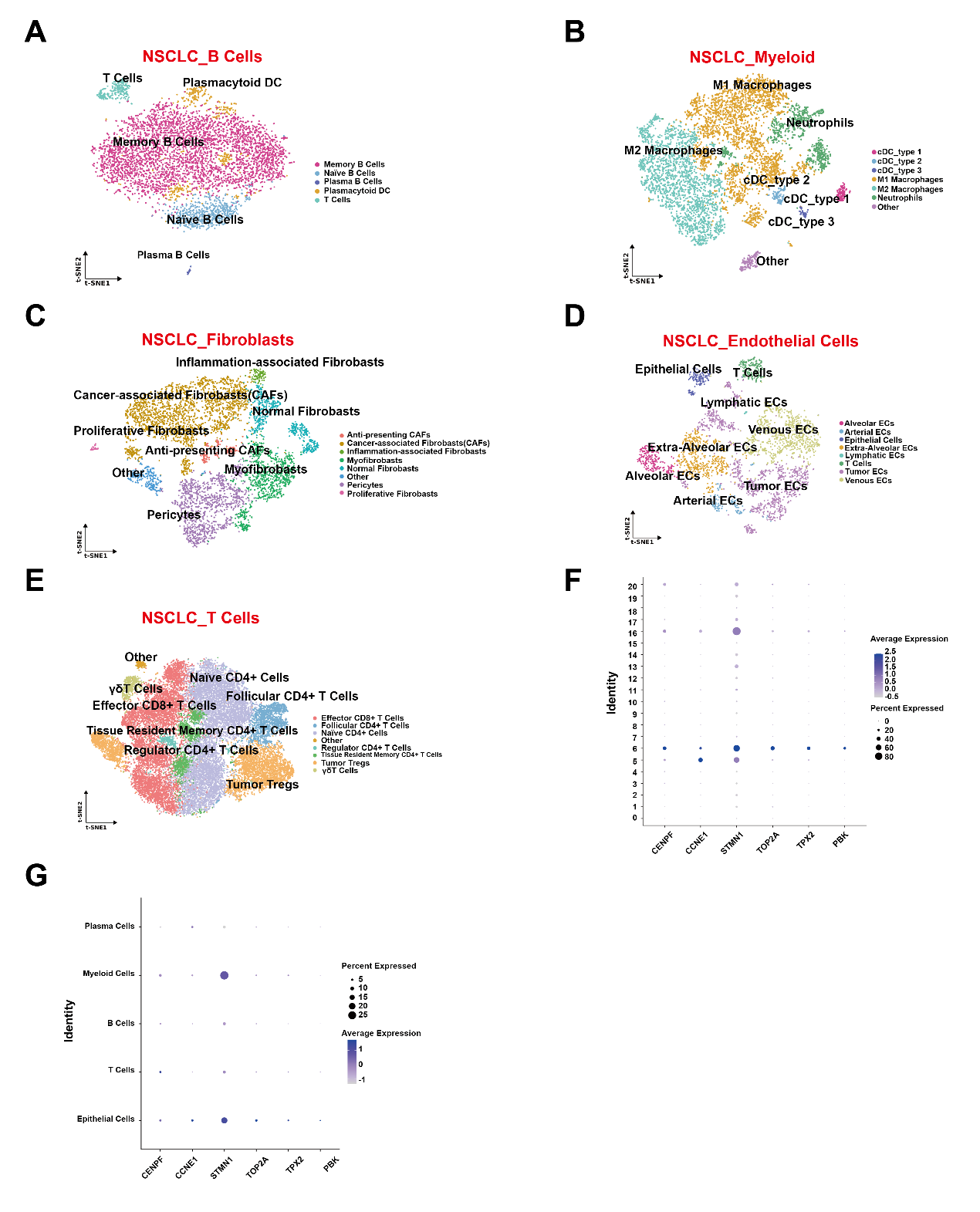


**Supplementary Fig. 1 Single-cell sequencing of each cell subpopulation cluster in NSCLC samples.**

Single-cell RNA sequencing (scRNA-seq) showing the subgroups of B **(A)**, myeloid **(B)**, fibroblasts **(C),** endothelial **(D)**, and T **(E)** cells in the NSCLC samples. **(F)** Cell markers of CCNE1(+) cells. **(G)** The expression analysis of CCNE1(+) in Epithelial cells and immune cells.


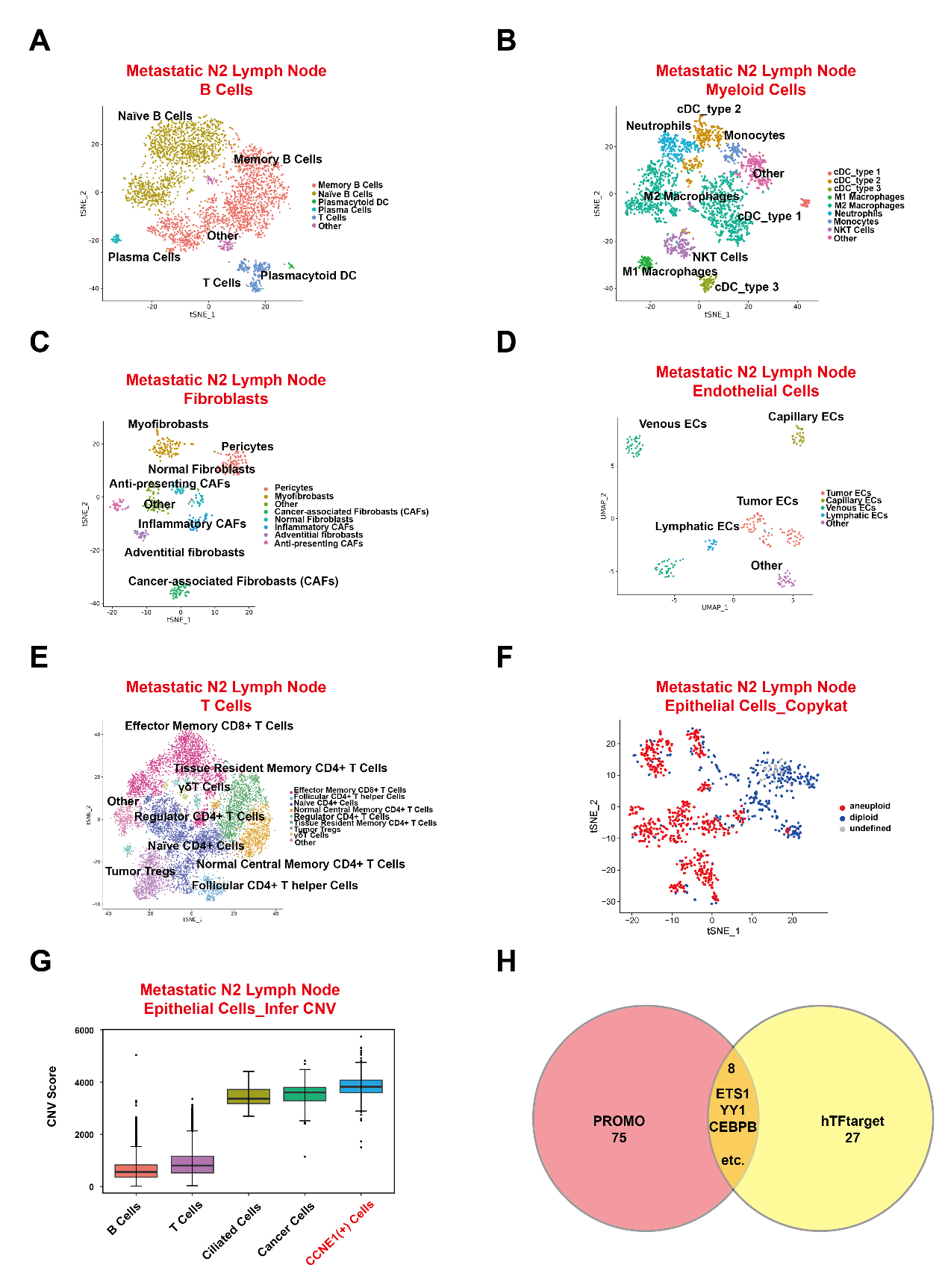


**Supplementary Fig. 2 Single-cell sequencing of each cell subpopulation clustering in metastatic N2 lymph node samples.**

Single-cell RNA sequencing (scRNA-seq) revealing subgroups of B **(A)**, myeloid **(B)**, fibroblasts **(C),** endothelial **(D)**, and T **(E)** cells in the metastatic N2 lymph nodes. **(F and G)** CopyCat and Infer Copy Number Variation analyses of subgroups of epithelial cells were performed to evaluate epithelial subgroup cell malignancies in metastatic N2 lymph nodes. **(H)** Database PROMOT and hTFtarget predicted that ETS1 might be a transcription factor.


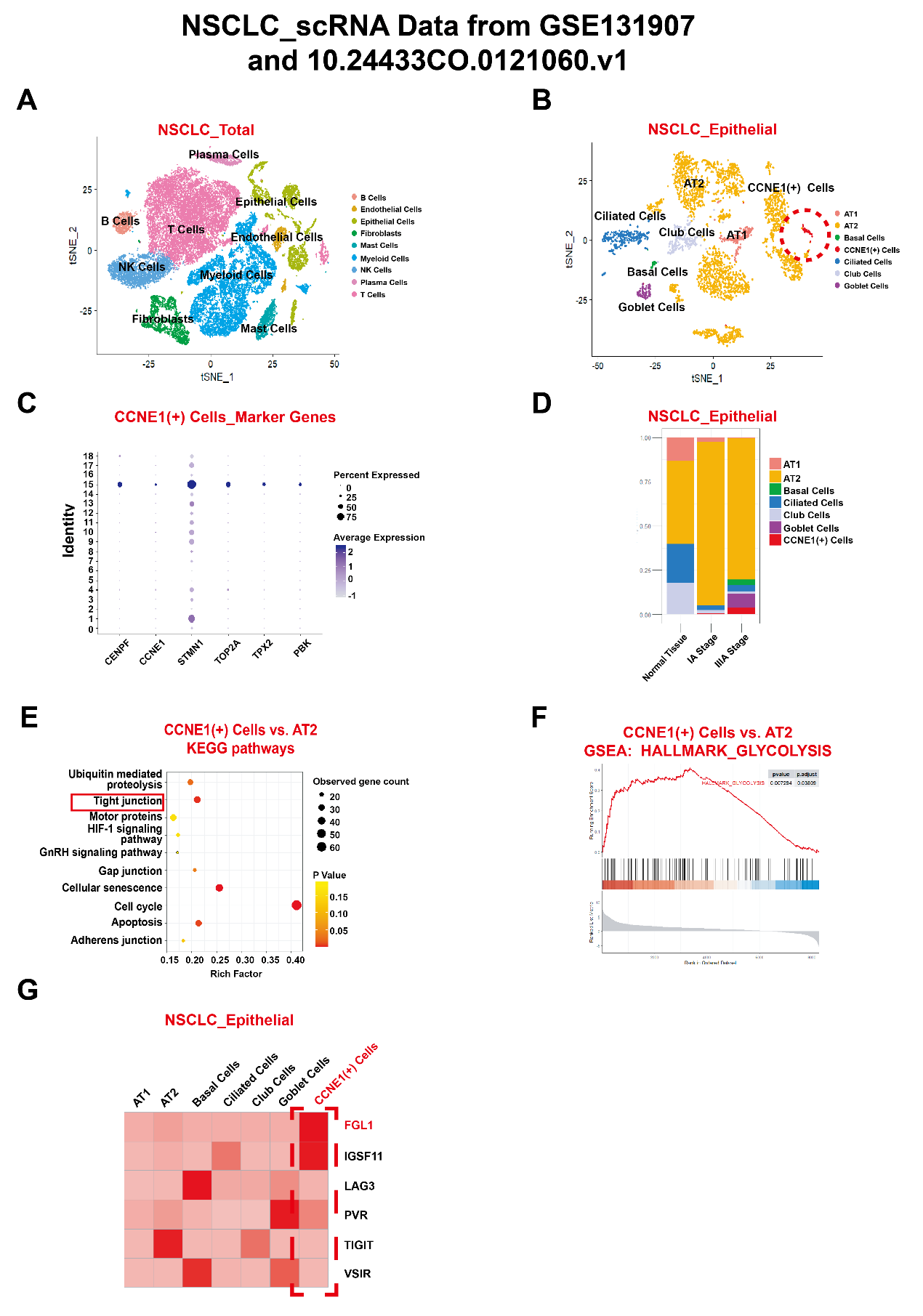


**Supplementary Fig. 3 The existence of CCEN1(+) cells in nine lung cancer-related samples was proven in the public dataset GSE 131907 and dataset 10.24433CO.0121060. v1.**

**(A)** Overall cluster map from scRNA-seq of nine samples. **(B)** Subgroup of epithelial cells in these samples. **(C)** The marker gene used to identify CCNE1(+) cells in our samples could also be used to identify this group of cells in public datasets. **(D)** The cell proportion analysis of Epithelial cells. **(E)** KEGG analysis was used to compare CCNE1(+) cells with AT2 cells, and differential gene analysis was performed. **(F)** GSEA was used to explore the role of CCNE1(+) cells based on the differentially expressed genes (DEGs) between CCNE1(+) cells and AT2 cells. **(G)** Various immune checkpoint gene expression levels (*FGL1*, *IGSF11*, *LAG3*, *PVR*, *TIGIT*, and *VSIR)* are displayed in a heat map in epithelial cells of NSCLC samples.


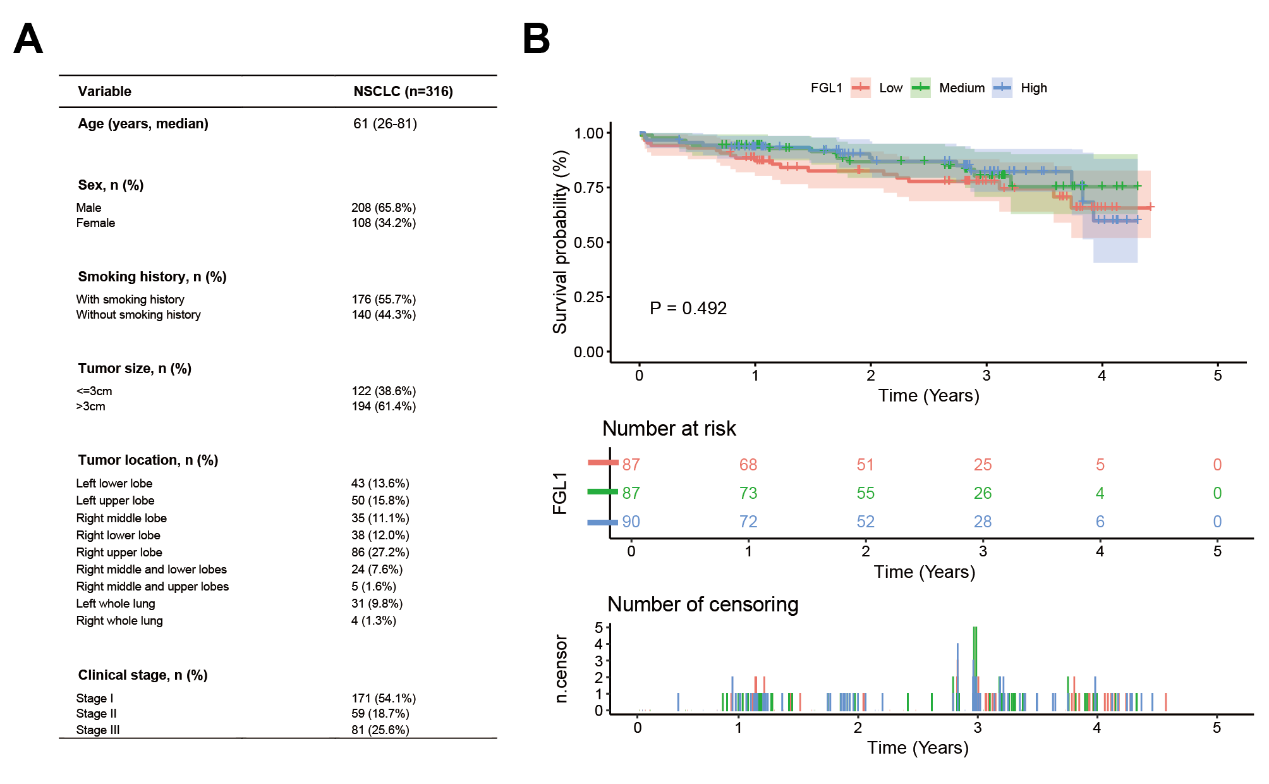


**Supplementary Fig. 4. The clinical characteristics of 316 NSCLC samples in tissue microarray and FGL1 related survival analysis.**

**(A)** The clinical characteristics of 316 NSCLC samples in tissue microarray. **(B)** Kaplan-Meier survival analysis revealed the association between FGL1 expression and overall prognosis.


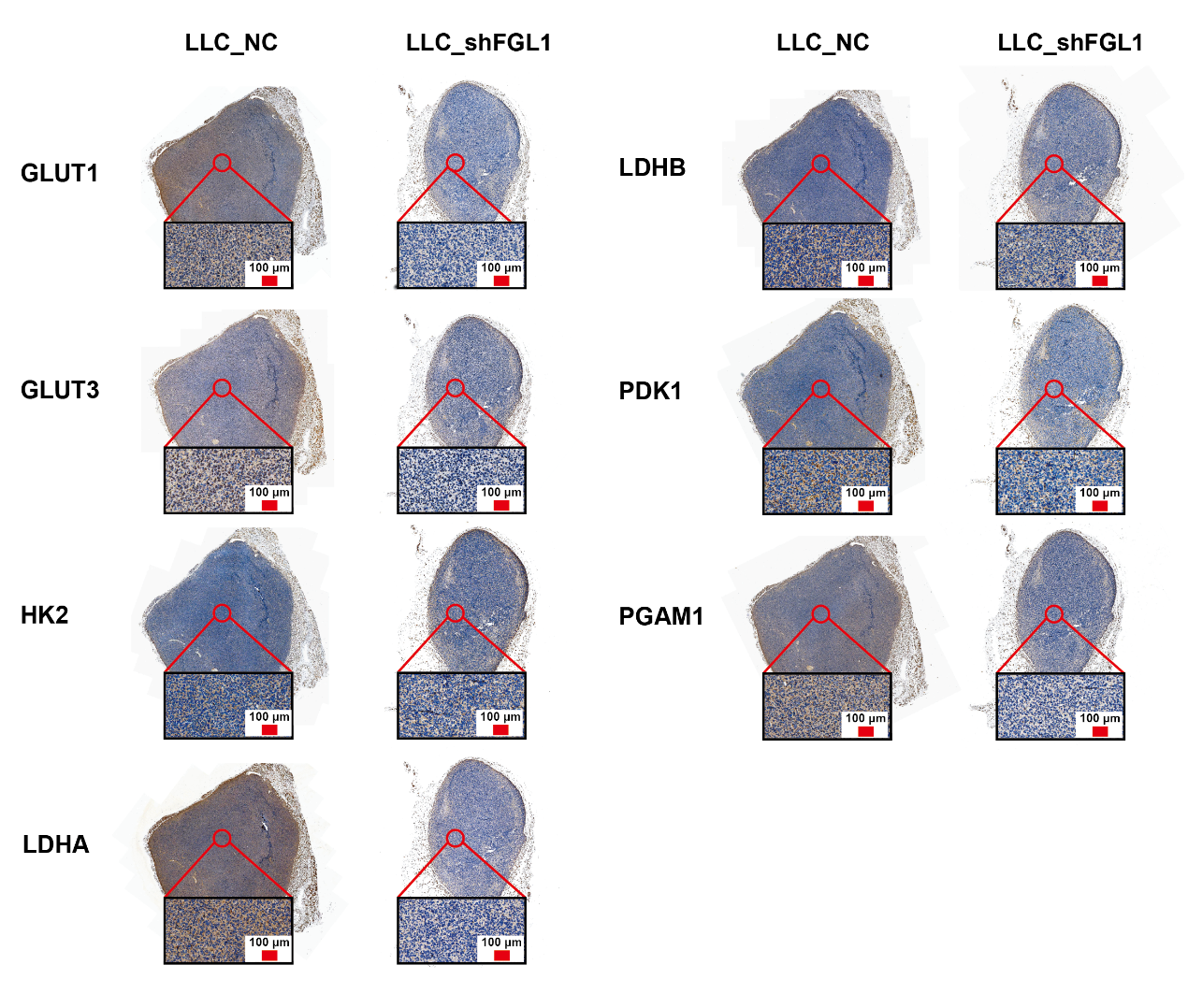


**Supplementary Fig. 5 IHC revealed that the expression of key glycolysis molecules was inhibited after FGL1 knockdown by lentivirus.**

Subcutaneous tumor-bearing nude mice showed that *FGL1* knockdown in LLC cells significantly inhibited tumor proliferation (Fig. 7A). IHC was performed to verify the changes in key glycolytic molecules after *FGL1* knockdown.


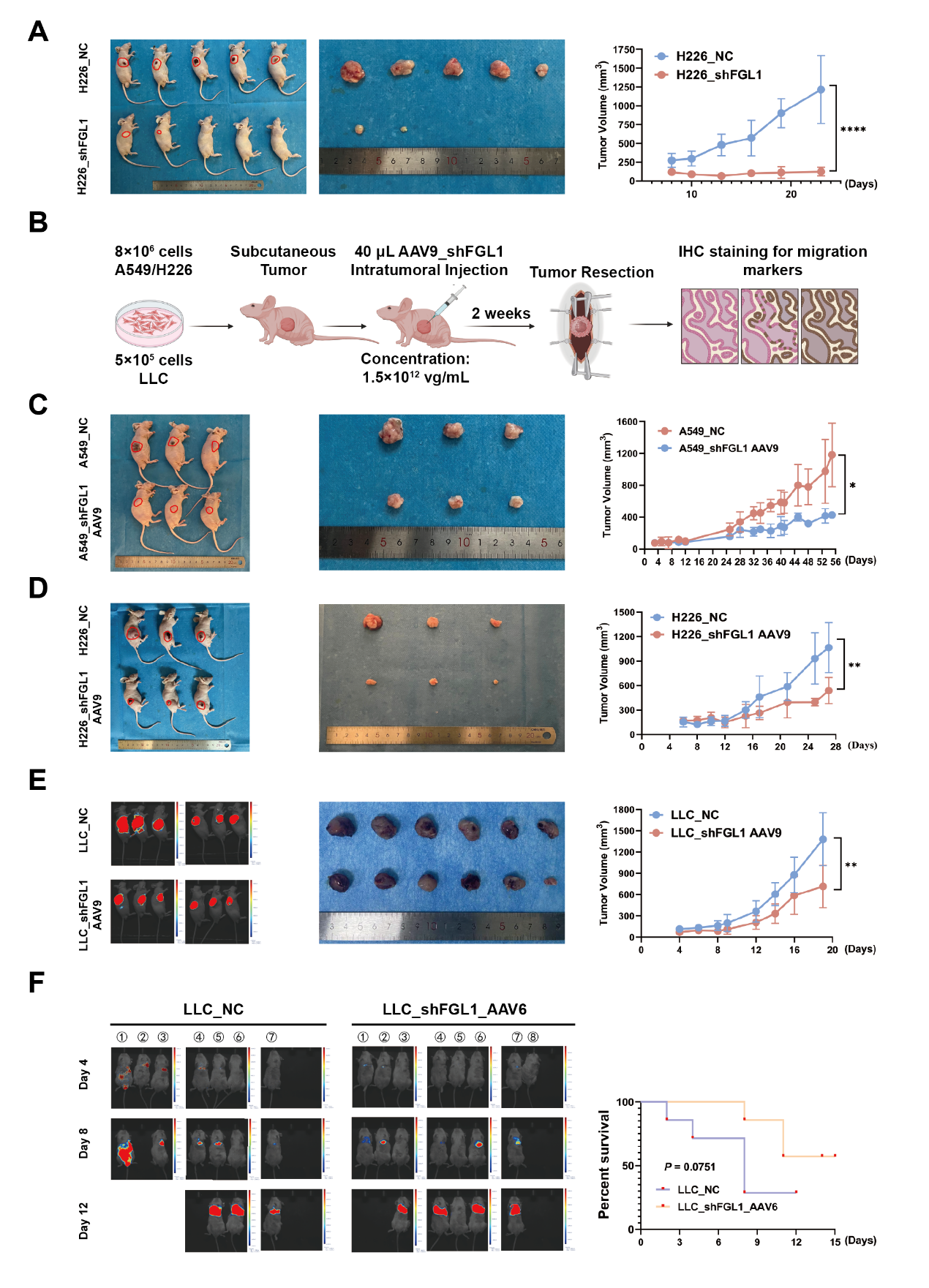


**Supplementary Fig. 6 shFGL1_AAV9 and shFGL1_AAV6 were constructed for FGL1 knockdown to inhibit lung tumor proliferation and metastasis in vivo.**

**(A)** Subcutaneous tumor-bearing nude mice showed that *FGL1* knockdown in H226 cells significantly inhibited tumor proliferation (n=10). **(B)** Schematic of the procedure for intratumoral injection of AAV9 to achieve FGL1 knockdown *in vivo*. **(C-E)** shFGL1_ AAV9 was constructed for intratumoral injection to achieve FGL1 knockdown in A549 (n=6), H226 (n=6), and LLC (n = 12) cell lines in vivo. **(F)** LLC was injected intrapulmonarily, followed by shFGL1_AAV6 administration via a noninvasive tracheal injection after three days. The therapeutic effect of shFGL1_AAV6 was observed using small animal imaging at 4, 8, and 12 days, and survival analysis was performed (n=15).
